# MicroRNA signatures differentiate types, grades, and stages of breast invasive ductal carcinoma (IDC): miRNA-target interacting signaling pathways

**DOI:** 10.1186/s12964-023-01452-2

**Published:** 2024-02-07

**Authors:** Vinod Kumar Verma, Syed Sultan Beevi, Rekha A. Nair, Aviral Kumar, Ravi Kiran, Liza Esther Alexander, Lekha Dinesh Kumar

**Affiliations:** 1 Cancer Biology, CSIR-Centre for Cellular and Molecular Biology, (CSIR-CCMB) Uppal Road, Hyderabad, Telangana 500007 India; 2 Department of Pathology, Regional Cancer Centre (RCC), Medical College Campus, Trivandrum, 695011 India

**Keywords:** Invasive ductal carcinoma, miRNAs, Breast cancer, Biomarkers, Signaling pathways

## Abstract

**Background:**

Invasive ductal carcinoma (IDC) is the most common form of breast cancer which accounts for 85% of all breast cancer diagnoses. Non-invasive and early stages have a better prognosis than late-stage invasive cancer that has spread to lymph nodes. The involvement of microRNAs (miRNAs) in the initiation and progression of breast cancer holds great promise for the development of molecular tools for early diagnosis and prognosis. Therefore, developing a cost effective, quick and robust early detection protocol using miRNAs for breast cancer diagnosis is an imminent need that could strengthen the health care system to tackle this disease around the world.

**Methods:**

We have analyzed putative miRNAs signatures in 100 breast cancer samples using two independent high fidelity array systems. Unique and common miRNA signatures from both array systems were validated using stringent double-blind individual TaqMan assays and their expression pattern was confirmed with tissue microarrays and northern analysis. In silico analysis were carried out to find miRNA targets and were validated with q-PCR and immunoblotting. In addition, functional validation using antibody arrays was also carried out to confirm the oncotargets and their networking in different pathways. Similar profiling was carried out in Brca2/p53 double knock out mice models using rodent miRNA microarrays that revealed common signatures with human arrays which could be used for future in vivo functional validation.

**Results:**

Expression profile revealed 85% downregulated and 15% upregulated microRNAs in the patient samples of IDC. Among them, 439 miRNAs were associated with breast cancer, out of which 107 miRNAs qualified to be potential biomarkers for the stratification of different types, grades and stages of IDC after stringent validation. Functional validation of their putative targets revealed extensive miRNA network in different oncogenic pathways thus contributing to epithelial-mesenchymal transition (EMT) and cellular plasticity.

**Conclusion:**

This study revealed potential biomarkers for the robust classification as well as rapid, cost effective and early detection of IDC of breast cancer. It not only confirmed the role of these miRNAs in cancer development but also revealed the oncogenic pathways involved in different progressive grades and stages thus suggesting a role in EMT and cellular plasticity during breast tumorigenesis per se and IDC in particular. Thus, our findings have provided newer insights into the miRNA signatures for the classification and early detection of IDC.

**Supplementary Information:**

The online version contains supplementary material available at 10.1186/s12964-023-01452-2.

## Background

Breast cancer is a heterogeneous disease and a major cause of mortality among women worldwide [1]. Ineffective utilization of expensive cancer screening methods and lack of diagnostic assays based on molecular markers for detecting the early and curable stages of breast cancer is the primary cause of mortality and poor survival among breast cancer patients in most developing countries [2, 3]. Breast cancer can be classified into different types based on its hereditary/receptor status [4] and into different grades and stages based on the extent of tumor progression. Current clinical diagnosis and classification of breast cancer rely on histological grading of the tumor and imaging techniques that are costly and tedious [5]. Therefore, it is prudent and need of the hour to have cost-effective, specific, and sensitive molecular diagnostic markers that can accurately detect the different molecular subtypes, grades, and stages, thus help in augmenting clinical management of this disease [6].

The use of gene expression profiling to determine the molecular classification of human cancers has recently gained impetus to discover novel biomarkers for diagnosis and prognosis of this disease [7–9]. The discovery of small non-coding RNAs in regulating gene expression has revolutionized clinical research, giving a new paradigm for creating and optimizing novel predictors of the disease status [10, 11]. MicroRNAs (miRNAs) are evolutionarily conserved, endogenous, small non-coding RNA molecules of about 22 nucleotides in length that function as modulators of gene expression [12, 13]. A scrutiny of literature from the last decade revealed the crucial role of miRNAs as oncomiRs and tumor suppressors, thereby demonstrating their role in tumor initiation and progression [14]. Accumulating evidence suggests the involvement of miRNAs in breast tumorigenesis and their aberrant expression has been exploited to serve as diagnostic, prognostic and therapeutic monitoring indicators [15, 16]. Delving into the research over the past years has substantiated the role of miRNAs in the molecular pathogenesis of breast cancer. Expression profiling datasets of breast cancer tissues have identified miRNAs that are aberrantly expressed in breast tumors, thus aiding in their classification. It has also been confirmed that miRNAs present in the body fluids termed circulating miRNAs open up new frontiers for developing non-invasive diagnostic and prognostic markers [17]. In terms of clinical value, the development of a liquid biopsy system based on these circulating miRNAs endows a promising strategy in developing next-generation biomarkers for the early diagnosis of cancer.

While recent progress suggests the potential of miRNAs as biomarker candidates for cancer diagnosis, prognosis, classification, and treatment planning, many studies suffer from insufficient sample size or lack of comprehensive validation. This makes it challenging to apply their findings broadly across diverse subjects, or to definitively establish miRNAs as reliable biomarkers. In order to address the lacuna in this research area, we have profiled the complete miRNA landscape (miRnome) of invasive ductal carcinoma samples (IDC), leveraging both TaqMan Low Density Arrays (TLDA) and Locked Nucleic Acid arrays (LNA). Our results provide robust evidence that this approach can accurately differentiate IDC by type, grade, and stage. MiRNA profiling was carried out using two different array platforms, and we subsequently validated our findings using TaqMan individual assays. This process led to the identification of 34 novel miRNAs specifically associated with human invasive ductal carcinoma. Furthermore, we investigated the specific role of these miRNAs as either oncogenes or tumor suppressors, employing in silico database development [18], followed by target validation through both shutdown and activation studies. Downstream assays like qRT-PCR, immunoblotting, and immunocytochemistry confirmed the regulation of the oncotargets by miRNAs. We further confirmed the overexpression of these biomarkers in various IDC samples through northern analysis and tissue microarrays from the same patient cohorts. In pursuit of an additional layer of target validation, we conducted antibody arrays, revealing promising avenues for directly connecting these miRNAs to specific oncogenic pathways. In order to perform in vivo validation, we deciphered the miRNA complement of the Brca2/p53 ^−/−^ mammary tumor model (Brca2/p53 double knockout murine model) [19] using TLDA (mouse arrays), identifying shared miRNAs between both species. To our knowledge, we are the first to report a set of validated novel and signature miRNAs, which could potentially serve as IDC biomarkers across specific types, grades and stages.

## Materials and methods

### Patient database and sample collection

A total of 100 IDC samples of different grades and stages along with their adjacent normal tissues [20] were collected (2-5 cm away from the designated malignant sample) in ‘RNAlater’ from women aged between 40 and 65 years (fulfilling the inclusion and exclusion criteria) who reported to Regional Cancer Centre (RCC), Trivandrum. Prior approvals were obtained from the ethical committees of both RCC and Centre for Cellular and Molecular Biology (CCMB), Hyderabad. All procedures were performed following the declaration of Helsinki, after obtaining written informed consent of the patients. After pathological examination and surgical intervention, they were segregated into different grades and stages and ascertained hormonal status (ER+/−; PR+/−) (Supplementary Fig. S1).

### Sample collection of animal model

Animal models were used strictly in accordance with the Committee for the Purpose of Control and Supervision on Experiments on Animals (CPCSEA) and the Institute’s animal ethical committees (IAEC) of CCMB. The breast cancer model (conditional Brca2/p53 knockout under control of Blg-cre transgene) develops autochthonous tumors on any of the 5 pairs of mammary glands in a 6- to 15-month time window. PCR conditions for genotyping of the Blg-cre transgene and the conditional alleles for Brca2 and p53 have been described [19]. A cohort of 5 untreated mice were taken for the study. Mammary tumors when reached with a size of ~1.2cm^3^ were dissected without any treatments after humane culling of animals and stored in RNAlater at − 80 degrees till experiments were carried out.

### Total RNA isolation and cDNA synthesis

Tissue samples (5-10 mg) were lysed using Qiagen Tissue lyser II and total RNAs were isolated using mirVana kit (ThermoFisherScientific, USA) after DNase treatment. The purity and quantity of RNA were measured using NanoDrop ND 1000 spectrophotometer (NanoDrop Technologies, USA) and integrity was checked by determining RNA integrity number (RIN) using an Agilent Bioanalyzer (2100). The cDNA was synthesized from total RNA (1 μg) using a High-capacity cDNA synthesis reverse transcription kit (Applied Biosystems, USA) as per the manufacturer’s instruction manual.

### MiRNA transfection

Breast cancer cell lines (MDA-MB 231, MCF-7) were seeded (1 × 10^5^ cells) into 24 well plate with 1 mL DMEM + 10% FBS with appropriate antibiotics (Day 0). When the cells reached 60–70% confluency, they were transfected with antimiRs and mimics of various miRNAs along with appropriate controls following the Thermo Fisher scientific protocols. They were then incubated for 24–72 hr. at 37 °C in a CO_2_ incubator and taken for confocal microscopy.

### TaqMan low-density array and locked nucleic acid array experiments

A total of 48 IDC samples consisting of 8 biological replicates from each of the biological groups were taken for both array analyses (Grade 2 and 3 (24 from each grade) consisting of 8 samples each from stages I, II III of every grade) and were used for normalizing with pooled 10 adjacent normal samples. TLDA (ver2.0), which contains 667 human miRNAs covering Sanger miRBase (ver10.0), was performed as per the manufacturer’s protocols. The experiments were repeated with LNA arrays (ver11.0) containing 1372 miRNAs from hmr-miRBase 14.0 + miRPlus from Exiqon, Denmark, following the manufacturer’s instruction manual. Rodent array (Applied Biosystems version v 3.0) consisting of 641 mouse and 373 rat unique miRNAs along with appropriate controls were carried out in 5 biological replicates of Brca2/p53 double knock out mammary models along with 6 wild type controls to find out the common miRNAs between human and rodent systems.

### TaqMan individual validation assays

Significant and valid human miRNAs that showed up/downregulation from TLDA and LNA arrays were subjected to double-blind TaqMan individual validation assays in 100 IDC samples (same 48 samples that has been used for array analysis along with an additional 52 samples) with appropriate control samples following manufacturer’s instructions and MIQE-guidelines.

### MiRNA target finding and validation by immunocytochemistry, q-PCR, immunoblotting, and northern blotting

In silico studies predicted putative miRNA targets which have been compiled in the form of a database oncomiRdbB [18]. The anti-miRNAs and mimics for miRNAs targeting breast cancer genes involved in key signaling pathways were purchased from Exiqon, Denmark and evaluated on their oncogenic targets through immunocytochemistry, q-PCR, and western blotting. Anti-miRNAs and mimics included hsa-miR-432, miR-662, miR-659, miR-105, miR-200c, miR-649 and miR-921 targeting caspase 8, c-Myc, Bcl2, APC, PTEN, p53, PAX5 and STAT3. For northern blotting, small RNAs were isolated using the mirVana kit and hybridization was done using the LNA miR-21 probe following the manufacturer’s protocol. ‘No-RT’ control was also set up to confirm the genomic DNA contamination.

### In-situ hybridization on tissue microarray

Tissue microarrays were made with 125 FFPE samples spotted on each slide, having three biological replicates from each group (Lab Surgpath-A Human Proteome Atlas group). In situ Hybridization (ISH) experiments were performed using miRCURY LNA miRNA Detection Probes designed for miR-21 following the manufacturer’s instructions. The images were scanned using the automated slide scanning system Scanscope XT (Aperio Technologies, Vista, CA), and the data was analysed.

### Antibody array

An antibody array allows for the screening/profiling of multiple proteins against the antibodies, which are immobilized and bound to the slide support through covalent interactions. In this study, proteins were isolated from 12 IDC samples each of stages I, II, and III in duplicates of both grade 2 and 3 along with their adjacent controls. All the proteins extracted from control and experimental samples were labelled with appropriate conjugate, followed by hybridization with an antibody array (Clontech, USA) consisting of 500 monoclonal antibodies related to several oncogenes and pathways spotted on a microscopic single glass slide. Unbound antibodies and free labels were removed by washing; then proteins were allowed to bind to antibodies on the array (membrane or glass). Once bound, the array was visualized as per the manufacturer’s instructions. Each array consisted of positive, negative, and internal controls to avoid redundancy in the number of antibodies-spotted on glass slides.

### Statistical analysis

Statistical analysis of the TLDA data set was carried out using the StatMiner (Spotfire) software from Integromics. The differentially expressed miRNAs were considered significant and valid for those with FDR adjusted *p-value* < 0.05. For LNA arrays, a two-tailed statistical t-test was performed among the sample groups with *p-values* lower than 0.001. The heat map was also made based on a cut-off of *p* < 0.001. The SD was mounted as error bars on histograms/line diagrams of each figure.

## Results

### Distinct miRNA signatures differentiate different grades and stages of IDC

**Fig. 1:**
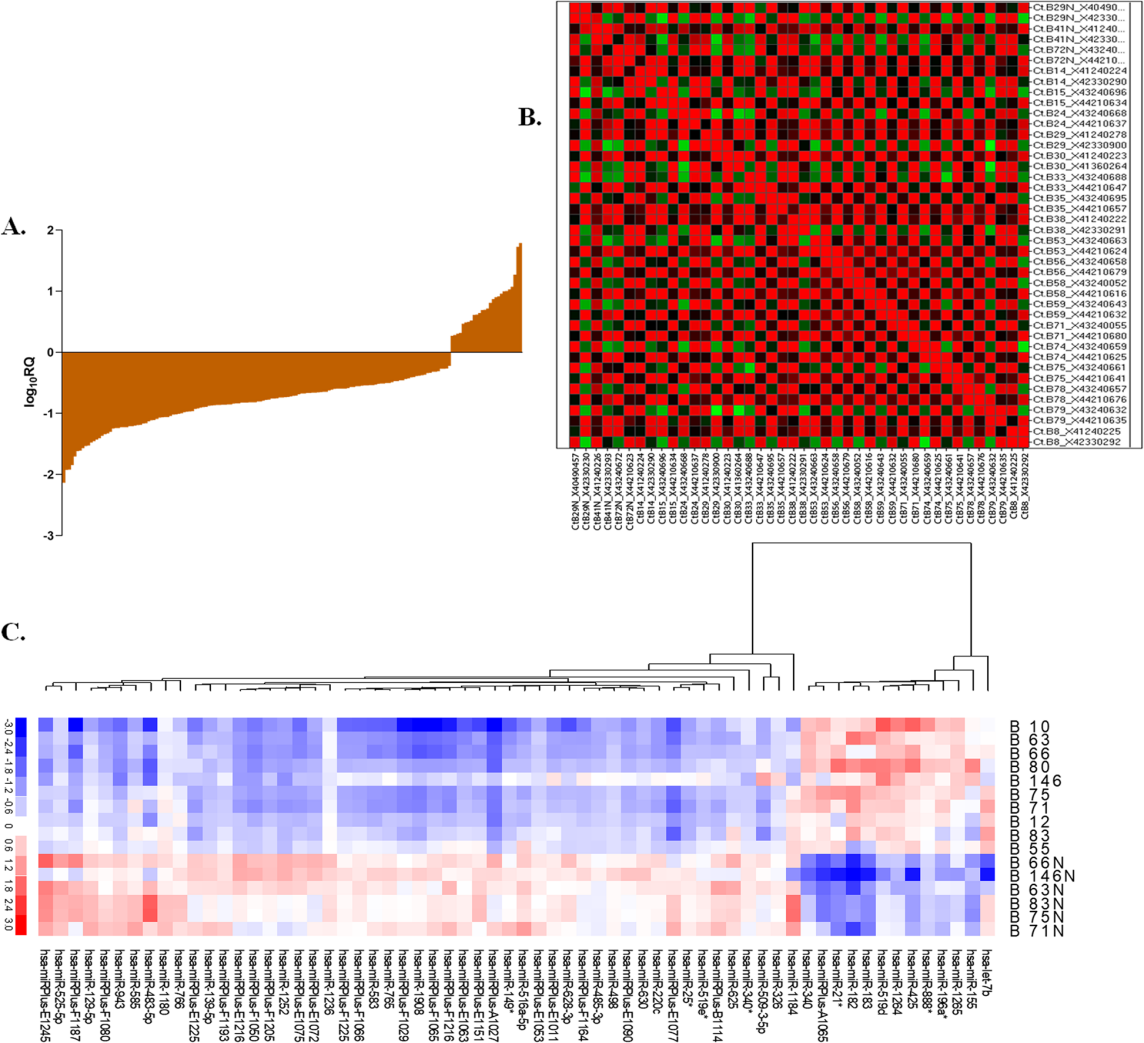
The expression patterns of miRNAs in IDC samples vs their adjacent normal samples (**A**) analyzed using TLDA and presented as fold change (log_10_ RQ), (**B**) TLDA heat map illustrates the gene expression profiles of carcinoma samples compared to their adjacent normal samples, with downregulated miRNAs depicted in red and upregulated miRNAs in green. (**C**) An additional heat map displays unsupervised hierarchical clustering of miRNAs expression in IDC samples vs adjacent normal as revealed by LNA arrays

Out of a total of 667 miRNAs from TLDA and 1372 from LNA arrays, 439 were detected as significant and valid for IDC samples compared to their adjacent normal samples. The general trend of expression of miRNAs in both the arrays showed 77% down-regulation and 23% up-regulation though there existed a marked difference between types, grades, and stages (Fig. 1A-C, Supplementary Fig. S2).

Different miRNA clusters were identified and analysed to find out the proximity between the miRNA families. MiRNAs with coordinated functions are often clustered together. To decipher the co-adaptation and functionality, miRNA cluster analysis was carried out and this revealed their co-ordinated expression pattern (Supplementary Fig. S3). Validation of significant miRNAs was carried out using double-blind TaqMan individual assays in 100 IDC samples, reconfirming our findings (Supplementary Fig. S4). *In-silico* studies (database-OncomirdbB) [18] further supported microarray data and identified 34 of them as novel breast cancer miRNAs as they were not reported earlier.

**Fig. 2:**
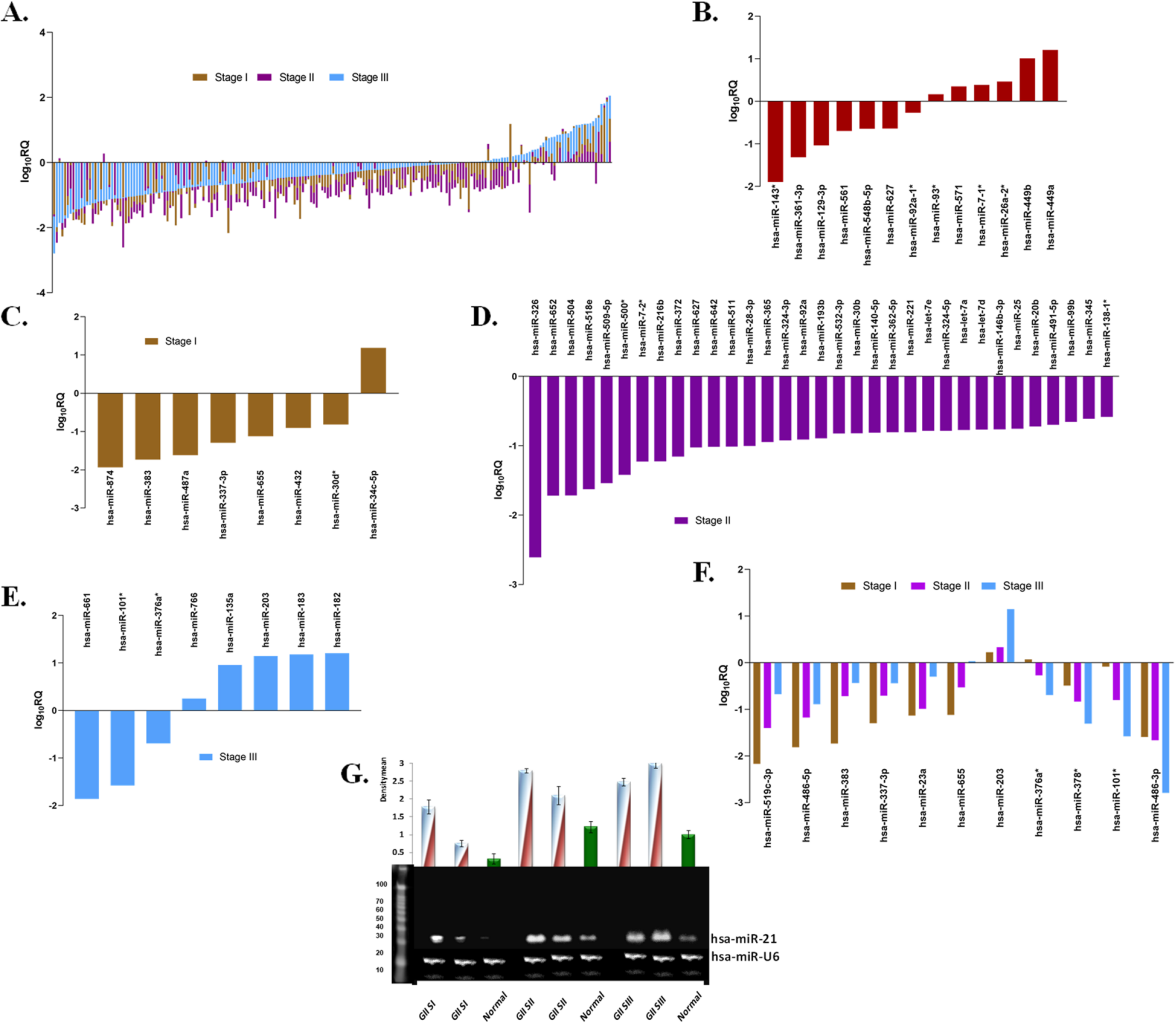
MiRNA expression profile of Grade 2 IDC samples vs their adjacent normal samples (**A**) Comparative trend of miRNAs in all stages of Grade 2 by TLDA represented as fold change, (**B**) unique and significant miRNAs in Grade 2, (**C**) unique miRNA signature in Stage I, (**D**) Stage II, (**E**) Stage III, (**F**) common miRNA signature reveals similarities between stage I, II and III of grade II, (**G**) Northern blot analysis showing an overexpression of miR-21 in different stages of grade 2 breast cancer when compared to adjacent normal samples

Grade 2 exhibited marked differences in the level of expression among its different stages (Fig. 2A). Thirteen miRNAs, hsa-miR-143*, hsa-miR-361-3p, hsa-miR-129-3p, hsa-miR-561, hsa-miR-548b-5p, hsa-miR-627, hsa-miR-92a-1*, hsa-miR-93*, hsa-miR-571, hsa-miR-7-1*, hsa-miR-26a-2*, hsa-miR-449b, and hsa-miR-449a, were unique and differentially expressed in grade 2 compared to grade 3 (Fig. 2B, Supplementary Fig. S5 and S6). Downregulated miRNAs of stage I included hsa-miR-874, hsa-miR-487a, and hsa-miR-30d* while the upregulated included hsa-miR-34c-5p (Fig. 2C). Likewise, unique ones of stage II and III were hsa-miR-509-5p, hsa-miR-365, hsa-miR-92a, hsa-miR-532-3p and hsa-miR-661, hsa-miR-376a*, hsa-miR-625* and hsa-miR-766 (Fig. 2D-E). MiRNAs common to all stages in grade 2 included significantly downregulated ones like hsa-miR-519c-3p, hsa-miR-486-5p, hsa-miR-383, and hsa-miR-101*, and the significantly upregulated hsa-miR-203 (Fig. 2F). The overexpression of miR-21 in all the stages of grade 2 compared to healthy individuals was further confirmed by Northern blot analysis (Fig. 2G).

**Fig. 3:**
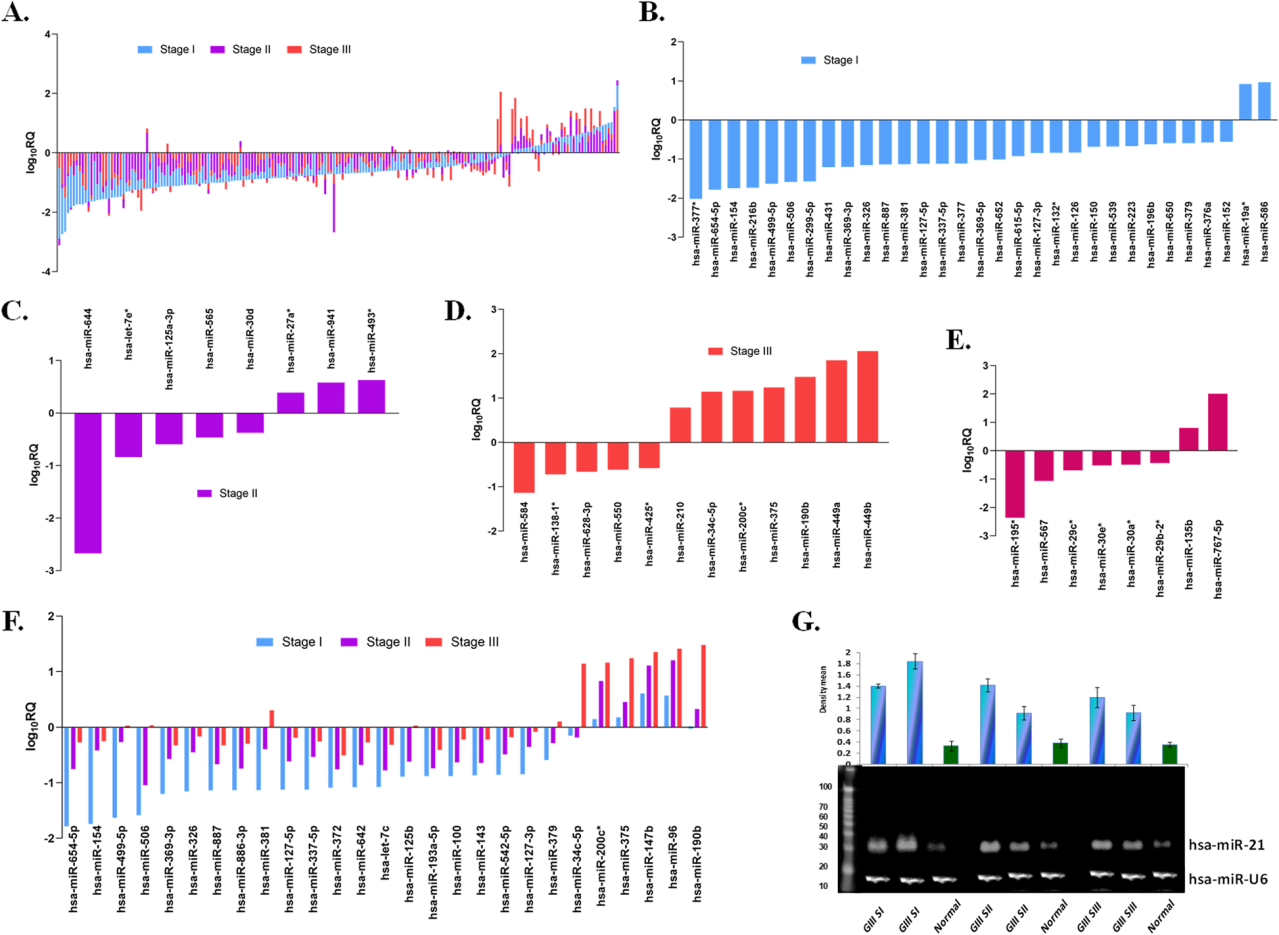
MiRNA signatures in Grade 3 IDC samples vs their adjacent normal samples (**A**) Differential expression trend of miRNAs in in all stages of Grade 3 vs adjacent normal samples by TLDA, (**B**) Significant miRNAs signatures in stage I, (**C**) stage II, (**D**) stage III, (**E**) unique and significant miRNAs in Grade 3, (**F**) common miRNAs between stage I, II and III, (**G**) Northern blot of miR-21 showing high expression in different stages of grade 3 as compared to adjacent normal cohorts

MiRNA expression pattern of grade 3 showed distinct variations among its stages (Fig. 3A, Supplementary Fig. S7). Out of 31 significant miRNAs found for stage I, 16 were novel that included notable miRNAs like hsa-miR-654-5p, hsa-miR-499-5p, hsa-miR-431, and hsa-miR-154 (Fig. 3B). On similar lines, in stage II, hsa-miR-493* and hsa-miR-941 showed overexpression, while hsa-miR-760 and hsa-let-7e* showed downregulated expression (Fig. 3C). Likewise, significant miRNAs for stage III comprised of hsa-miR-584, hsa-miR-138-1*, hsa-miR-210, hsa-miR-220c*, and hsa-miR-449b (Fig. 3D). Eight miRNAs were novel and specific for grade 3 and not detected in grade 2 (Fig. 3E). Interestingly, among the common ones at the same cut-off value, hsa-miR-381, hsa-miR-34c-5p, and hsa-miR-379 showed opposite expression patterns in stage III of grade 3 in IDC (Fig. 3F). Moreover, a gradation of miR-21 expression was observed in all stages of grade 3, with the highest expression in stage I (Fig. 3G).

### Differential expression of miRNAs in ER + and ER – subtypes

**Fig. 4:**
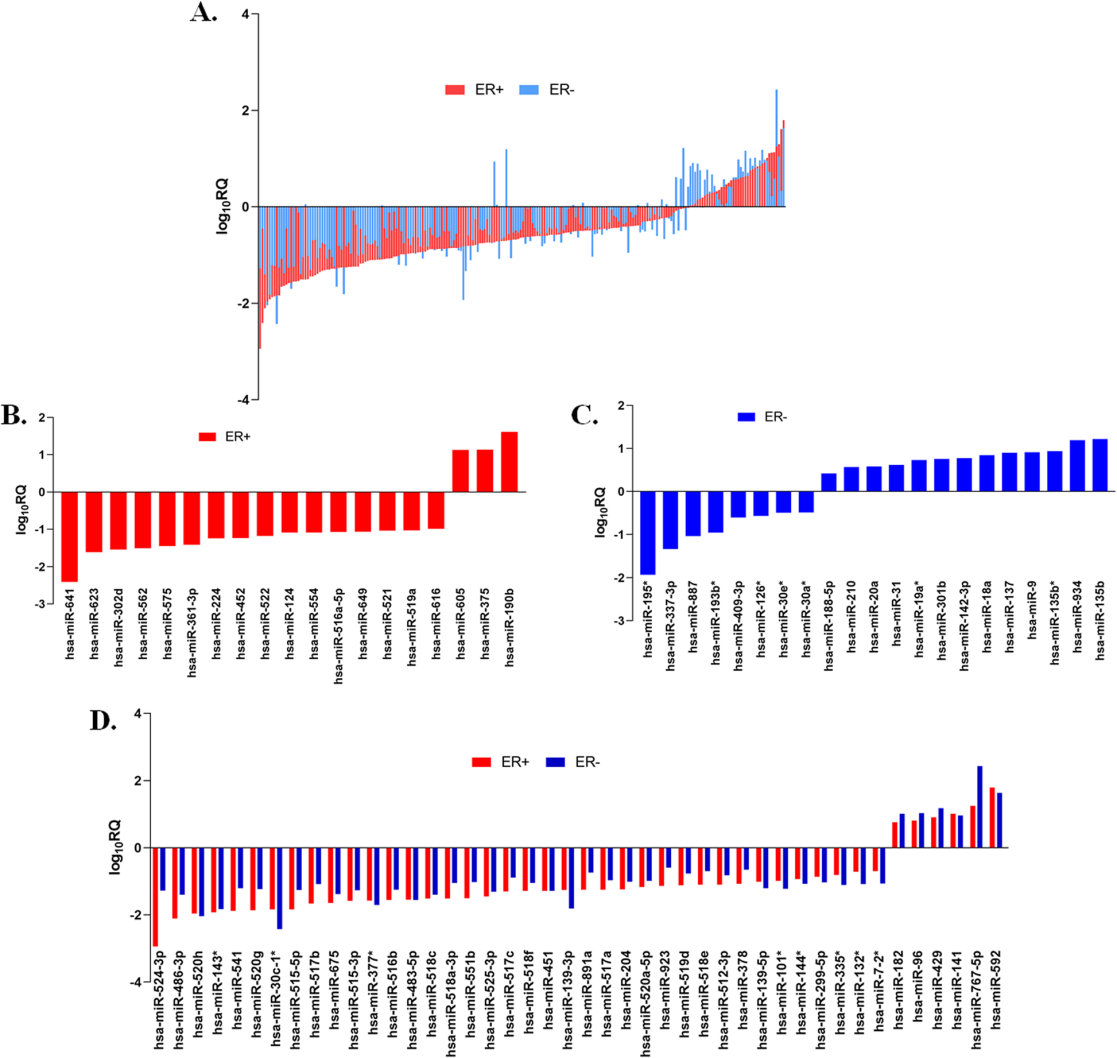
Differential expression of miRNAs in ER subtypes of IDC samples vs their adjacent normal samples (**A**) Differential trend of miRNAs expression was observed between ER+ and ER- samples in TLDA analysis (**B**) Significant miRNAs in ER+, (**C**) ER-, and (**D**) common and significant miRNAs in both the subtypes were analyzed with the *p* < 0.05 using Graphpad 8.0

About 80% of the breast cancer cells grow in response to hormone estrogen and hence they are classified as ER + and ER-. These subtypes of breast cancer showed unique expression patterns of miRNAs (Fig. 4A, Supplementary Fig. S8). Analysis of ER + showed a set of 19 such uniquely expressed miRNAs which included downregulated ones such as hsa-miR-641, hsa-miR-623, hsa-miR-562 etc., and upregulated ones like hsa-miR-375, hsa-miR-605, and hsa-miR-190b (Fig. 4B). Similarly, at the same cut-off value, 21 miRNAs such as hsa-miR-887, hsa-miR-188-5p, hsa-miR-301b, hsa-miR-9 and hsa-miR-142-3p etc. were unique to ER- (Fig. 4C). Among the miRNAs that showed common expression profiles in both ER+ and ER- subtypes, 38 of them were down-regulated and 20 exhibited upregulation. (Fig. 4D). Some of the upregulated miRNAs included hsa-miR-592, hsa-miR-141, hsa-miR-429, hsa-miR-96, hsa-miR-182, hsa-miR-767-5p, has-miR-142-3p and the downregulated ones were hsa-miR-524-3p, hsa-miR-486-3p, hsa-miR-520 h, hsa-miR-143*, hsa-miR-541, hsa-miR-520 g, hsa-miR-515-5p, hsa-miR-675, hsa-miR-516b.

### In silico identification and in vitro validation of miRNAs targets

**Fig. 5:**
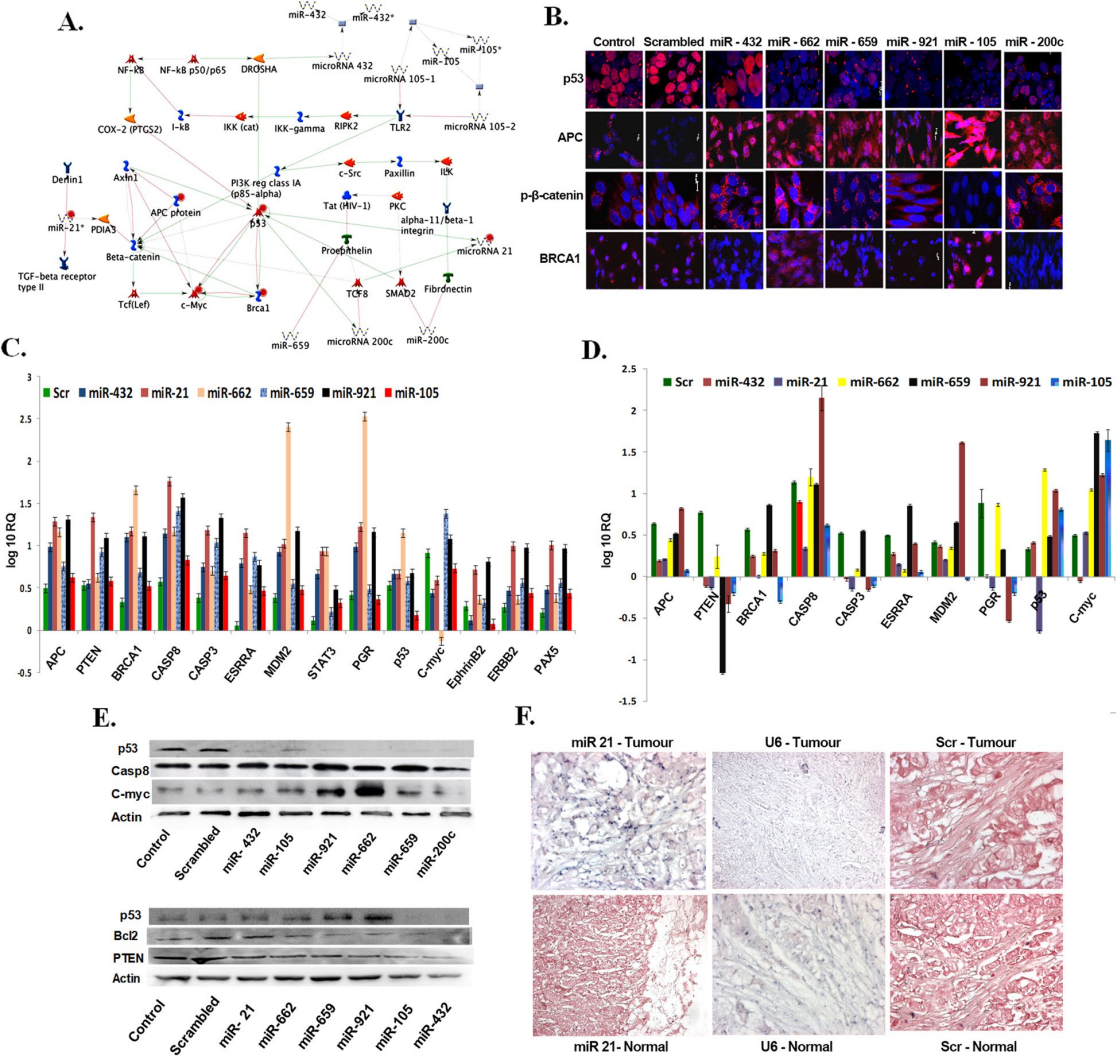
*In-silico* prediction and target validation using mimics and antimiRs (**A**) Analysis by GeneGo pathway reveals oncogenic targets for the respective miRNAs, (**B**) Immunocytochemistry performed in MDA-MB-231 breast cancer cell by mimics of miR-432, miR-662, miR-659, miR-921, miR-105, and miR-200c and observed their effects on targets p53,APC, p-β-catenin and BRCA1, (**C**) q-PCR analysis using mimic probes of miR-432, miR-21, miR-662, miR-659,miR-921,and miR-105 demonstrating an induction effect on targets APC, PTEN, BRCA1, CASP8, CASP3, ESRRA, MDM2, STAT3, PGR, p53, c-Myc, EphrinB2, ERBB2, and PAX5 (**D**) varied effects on respective targets using KD probes of miR-432, miR-21, miR-662, miR-659, miR-921 and miR-105, (**E**) Upper panel denotes immunoblots on targets p53, caspase 8, c-Myc, using mimics of miR-432, miR-105, miR-921, miR-662, miR-659, and miR-200c and lower panel denotes immunoblots on targets p53, Bcl2, and PTEN using KD probes of miR-21, miR-662, miR659, miR-921, miR-105 and miR 432 (**F**) In-situ hybridization in tissue microarrays of both tumor (upper panel) and its adjacent normal tissues (lower panels) using miR-21, U6 and Scr (scrambled) probes respectively

The database oncomiRdbB [21] was created and extensive pathway analysis through KEGG and Gene GoMetaCore program were used to explore the interaction between their putative targets (Fig. 5A, Supplementary Fig. S9, Supplementary Table S1). Targets were selected based on up/down regulation status of microRNAs and further validation was performed using miRNA mimics and knockdown (KD) probes for a range of oncotargets like p53, p-β-catenin, PTEN, BRCA1, CASP8, CASP3, Bcl2, ESRRA, MDM2, STAT3, PGR, c-Myc, EphrinB2, ERBB2, PAX5 and APC (Fig. 5 B-E). Immunocytochemistry showed that mimic probes of miR-662, miR-659, miR-921, miR-105, and miR-200c downregulated p53 while no change was observed with miR-432; however, they all could activate the APC, a tumor suppressor gene with an overexpression observed with miR-105. Mimics of miR-432, miR-662, miR-659, and miR-200c induced a cytoplasmic accumulation of p-β-catenin in cancer cells, while miR-105 brought down p- β-catenin to subnormal levels in the cytoplasm. However, miR-921 did not show any change with respect to scrambled and control cells (Fig. 5B; Supplementary Fig. S10). Transcript levels were assessed by quantitative PCR using mimics and knockdown probes on 15 plausible oncogenic pathway moieties as described above. Mimic probes of miR-432, miR-21, miR-662, miR-659, miR-921, and miR-105 showed an induction effect on all the targets (Fig. 5C); while KD probes of the same exhibited shut down in some targets while the inductive effect on others (Fig. 5D). These results clearly established that the selected/examined miRNAs acted as either positive or negative regulators of their respective targets, which were further corroborated by the immunoblotting studies (Fig. 5E, Supplementary Fig. S11). In-situ hybridization (ISH) of miR-21 along with the positive control (U6) and negative control (scrambled-Scr) on tissue microarray made from the same patient samples confirmed its overexpression in these tumor tissues (upper panel) compared to their respective adjacent normal tissues (lower panel) (Fig. 5F). Antibody array analysis of grade 2 and grade 3 samples established the level of expression of significant targets and their networking in multiple cancer pathways (Supplementary Fig. S12, Supplementary Table S2).

### Common miRNAs between human and Brca2/p53^(−/−)^ murine models

**Fig. 6:**
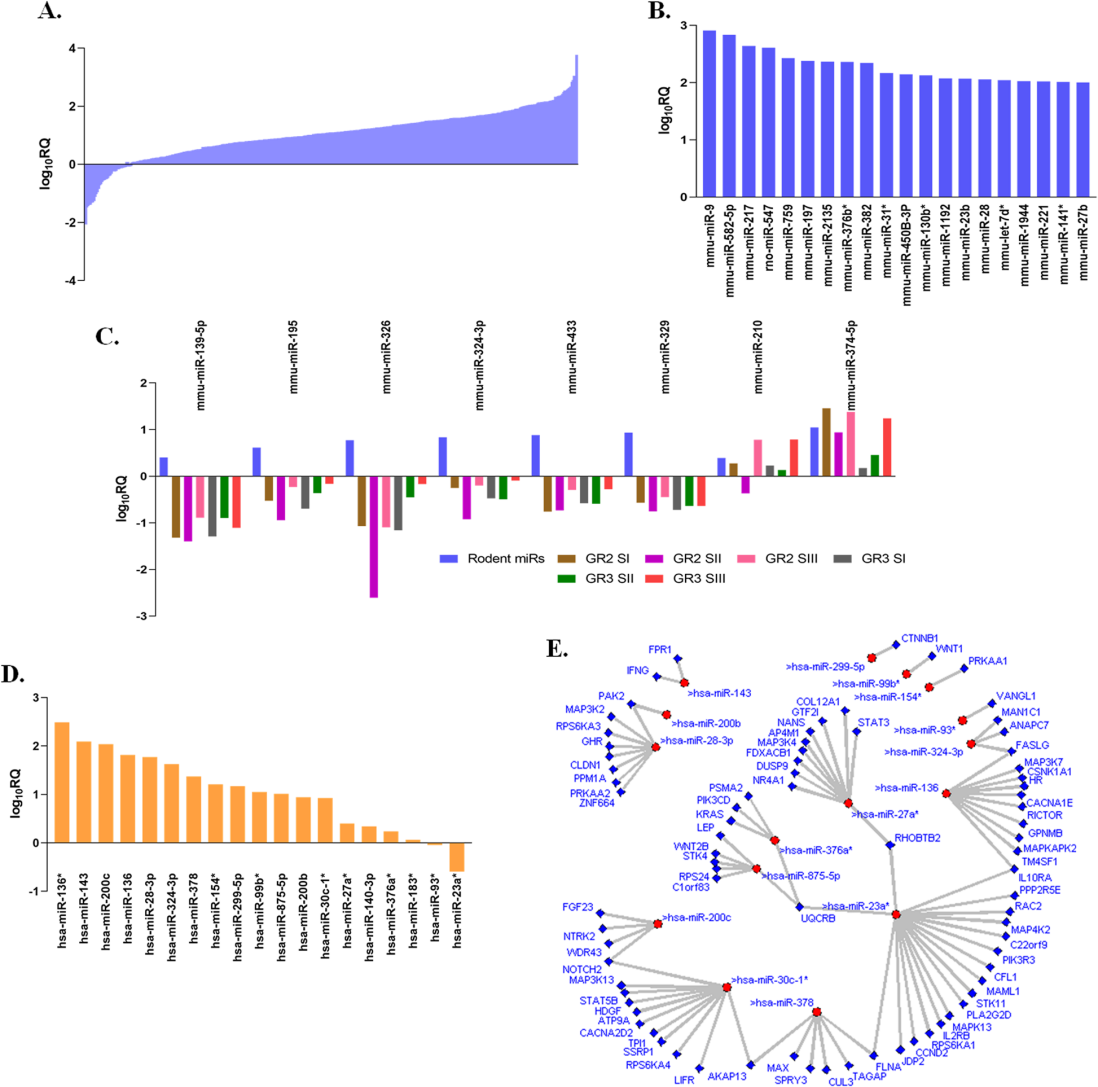
MicroRNA signatures of murine Braca2/p53 knockout models (**A**) The expression trends of murine miRNAs were analyzed using Rodent Array (TLDA), (**B**) Unique and significant miRNAs, along with their respective fold changes, were identified, (**C**) Common miRNAs between rodent and human breast cancer samples of different grades and stages (**D**) Human breast cancer miRNAs displaying significant expression in the murine system were identified, (**E**) Common miRNAs and their target interactions were elucidated, with miRNAs represented as red circles and targets depicted as blue square boxes

The double-knock out Brca2/p53^***−/−***^ murine models under the control of Blg-cre transgene developed autochthonous mammary tumors in about 6–15-month time frame. These tumors can occur in any of the 5 pairs of mammary gland [19]. MiRNA expression profiling of these mammary models showed a distinct and varied trend of expression compared to humans (since such a knock out condition does not exist in humans) (Fig. 6A). Unlike the human profile, where the majority of miRNAs were down-regulated, 80% were up-regulated in these mouse models. Among the murine miRNAs, mmu-miR-9, mmu-miR-582-5p, mmu-miR-197, mmu-miR-130b*, and mmu-let-7d were among the most significant and upregulated ones (Fig. 6B). Out of 78 human miRNAs spotted along with rodent ones, 20 of them were found to be significant and common between the two species (Fig. 6C). Murine miRNA, mmu-miR-374-5p was noted to have similar fold-change as human miRNAs, whereas others showed the inverse correlation. Human hsa- miR-23a*, hsa- miR-93*, hsa- miR-183*, hsa- miR-376a*, hsa- miR-875-5p, hsa- miR-154*, hsa- miR-378, hsa- miR-324-3p, and hsa- miR-136* displayed significant expression levels in murine system (Fig. 6D). Network analysis of these miRNAs showed common putative oncotargets involved in breast cancer initiation and progression (Fig. 6E). The common microRNAs from both human and mice models could be used for future in-vivo validation studies (Supplementary Table S3).

## Discussion

Identifying reliable biomarkers for early and affordable diagnosis of human disease is an area of extensive investigation. According to GLOBOCAN 2020, breast cancer has surpassed lung cancer to become one of the leading cancers worldwide. Though the current treatment modalities have marginally increased the patients’ overall survival, early diagnosis that is critical for effective treatment and prognosis, remains elusive due to ineffective or expensive screening methods. MiRNAs are considered to be master regulators of our genome and hence expected to control a vast majority of gene expression [22, 23], Many studies have implicated the promising potential of miRNAs as diagnostic and prognostic indicators for evaluating disease status and therapy monitoring [24, 25]. However, to classify them as effective biomarkers, research demands an in-depth validation of their occurrence and functionality. Therefore, in our study we undertook a comprehensive miRNA profiling of IDC samples. By analysing different types, grades, and stages using two distinct array platforms, we identified 439 miRNAs associated with breast cancer. Unsupervised hierarchical clustering and molecular characterization clearly distinguished specific and common miRNA signatures. Among these, 107 miRNAs qualified to be potential biomarkers for detecting different types, grades, and stages of this breast cancer after stringent TaqMan individual assays (Supplementary Table S4). They were further confirmed by northern analysis and tissue microarrays in the same set of samples. In addition, *in-silico* analysis of the pathways affected by the miRNAs along with in-depth target validations using antibody arrays confirmed the involvement of these miRNAs in various oncogenic pathways. For facilitating in vivo validation experiments in the future, we have also carried out rodent miRNA profiling in Brca2/p53^(***−/−)***^ mammary models. We found the expression patterns to be opposite to those in humans. This discrepancy could be due to differences in tumor origin between the two species. Furthermore, our reported miRNAs in rodent systems were in concordance with the miRNAs reported in different mammary tumor models [26]. Thus, the above-mentioned comprehensive validation studies provided insights into miRNA-oncotarget interactions, supporting our interpretation to establish these miRNAs as biomarkers for better management of IDC.

Various profiling studies have elucidated miRNAs as ideal biomarker candidates for breast cancer diagnosis [17, 27, 28]. Altered expression of various miRNAs already reported in different studies of breast cancer was in accordance with our findings [29, 30]. The miRNAs we have identified as candidate biomarkers have been validated to be involved in classification of breast cancer in other studies as well [29, 31]. This comparison revealed that subtype specific miRNA biomarkers could be used for a more precise disease classification [32]. Distinct and commonly deregulated miRNA signatures such as hsa-miR-21 in various types, grades and stages of IDC of our tissue microarrays (TMAs) are in concordance with various other studies, [33, 34] thus helped in understanding its regulatory role in the initiation and progression of breast cancer. Some of the identified novel miRNAs downregulated in our study have been previously reported to be associated with other cancers as well [35, 36]. Another study has confirmed that miR-375 actually repress the viability, migration, and invasion of MCF-7 breast cancer cell lines by targeting the expression of PAX6 [37]. Similarly, Elgamal et al. have demonstrated that lower levels of miR-205 might function as a tumor suppressor by inhibiting proliferation and invasion of cancerous cells by targeting HMGB3 [38]. In support of our findings, miR-20a, identified as an overexpressed miRNA in our study, has been shown to negatively regulate the induction of angiogenesis in MCF-7 cells [39].

Hsa-miR-361-3p, downregulated in our study, was reported to act as a tumor suppressor gene in non-small cell lung carcinoma (NSCLC) [40]. Hsa-miR-136 and hsa-miR-143* family found in the present study was earlier reported to supress proliferation, migration, and invasion in osteosarcoma patients [41, 42]. Hsa-miR-561 displayed in this profiling study, demonstrated inhibition of cell proliferation and invasion by downregulating c-Myc expression in gastric cancers in a previous study [43]. However, the overexpression of hsa-miR-34c-5p exhibited in stage I of grade 2 tumors of our study, are found to have a protective role in lung cancer cells from chemotherapeutic drug paclitaxel-induced apoptosis, proving it to be anti-apoptotic [44]. Although these miRNA signatures were found to be informative for different tumor types, grades, and stages, their extensive functional network remains to be elucidated.

To this end, we integrated miRNA target prediction with its functional annotation to build an overview of miRNA regulatory networks involved in breast cancer tumorigenesis. Some of the microRNAs showed specific and significant up/down regulation in certain grades and stages but not in others and hence taken ahead for downstream validation assays so as to identify them as biomarkers. For this purpose, we selected a few miRNAs like miR-432, miR-662, miR-659, miR-921, miR-105, miR-200c, miR-21, and designed mimics and anti-miRNAs (KD probes) for functional experiments. Immunocytochemistry, immunoblot analysis, and KEGG pathway enrichment with in-vitro validation, indicated a dynamic interaction between the differentially expressed miRNAs and their identified targets. The mimics of miR-662, miR-659, miR-921, miR-105 and miR-200c downregulated p53 that is corroborated by immunoblotting. An increase in APC levels were observed while transfecting with all mimics, however, miR-432, miR-662, miR-659, and miR-200c led to an accumulation of cytoplasmic phospho β-catenin, while miR-105 brought it down to subnormal levels. The significant increase in APC along with cytoplasmic phopho β-catenin levels has been correlated to have an active role in cell-cell matrix interactions [45]. Though the correlation between the increased APC and phospho β-catenin levels by certain miRNAs indicated a reduced Wnt signalling state [46], the inverse correlation of the same by miR-105 cannot be associated with Wnt deregulation. This suggests that other components of the Wnt pathway may also be altered resulting in different capacity of β-catenin to be regulated by APC that needs further investigation. In another study, hsa-miR-432 has been shown to act as a tumor suppressor gene by targeting LRP6, TRIM29, and Pygo2, thus deactivating the Wnt pathway [47]. Knockdown of miRNAs such as miR-662 and miR-659 led to an increased expression of all the targets suggesting an oncogenic role of these miRNAs [48]. Similar observations have been made in another study, where overexpression of miR-662 was shown to increase invasiveness and chemoresistance in non-squamous lung carcinoma potentiating its role as an oncogene. Hsa-miR-498 was revealed to downregulate BRCA1, one of the pivotal genes involved in breast cancer progression signifying its role in therapeutics [49].

Many of the miRNAs mentioned in this study are known to be linked to EMT and tumor plasticity. These include hsa-miR-9 (grade 3-stage I), hsa-miR-10b (grade 3-stage I), hsa-miR 34-a, (grade 2-stage I) hsa-miR-143 (grade 2, grade 3-stage I,II,III), hsa-miR-155 (grade 2-stage II and grade 3-stage III), hsa-miR-200c (grade 3-stage III), hsa-miR-203 (grade 2- stage III), hsa-miR-365 (grade2-stageII), and hsa-miR-661 (grade2-stageIII). Among these, few have been known to accelerate EMT for instance, Wang et al. showed that E-cadherin is a direct target of miR-9 and its upregulation had led to a consecutive downregulation of the expression of E-cadherin in NSCLC tissues [50] Further, miR-10b have been seen promoting EMT and invasion by targeting HOXD10, a transcription factor that acts as a suppressor of EMT [51]. On the contrary, miR-34a inhibits EMT by targeting Snail, a transcription factor that promotes EMT [52]. Zhai et al. demonstrated that the inverse correlation between miR-143 and its target ERK5 in breast cancer tissues led to suppression of GSK-3β/Snail signaling induced EMT [53]. MiR-155 is upregulated in various malignancies and a study carried out by Liu et al. demonstrated that downregulation of miR-155 minimized EMT processes in MCF-7 cells indicating the promotive role of miR-155 in EMT [54]. The miR-200 family is known to hinder EMT progression by repressing the expression of transcriptional repressors ZEB1 and ZEB2, which are known suppressors of E-cadherin [55]. The data published by Perdigão–Henriques et al. proposed that miR-200c inhibits vimentin and α-SMA expression and diminishes the nuclear translocation of β-catenin, suggesting that it suppresses EMT processes. Furthermore, their study indicated that miR-200c exercises a role in promoting the epithelial phenotype by stabilizing actin filaments in lamellipodia and filopodia [56]. It was shown by Huang et al. that miR-203 inhibits SMAD3 in TGF-β-induced EMT progression and invasion of NSCLC cells by interacting with particular regions of the 3′-UTR of SMAD3 [57]. The overexpression of miR-365 was found to enhance the expression level of E-cadherin thereby, suppressing the expressions of Vimentin and N-cadherin in NCI-H1975 cells thus, impeding the EMT process [58]. Significant downregulation of epithelial markers and upregulation of mesenchymal markers was observed when miR-661 was overexpressed, whereas the inhibition it generated opposing effects in NSCLC cells, A549 and SPC-A-1 [59]. These associations of miRNAs with the process of EMT and plasticity have enlightened researchers about the intricate network of miRNA molecules that connect the numerous pathways involved in cancer alone. Also, antibody arrays helped to correlate our findings of miRNA target interactions and helped to elucidate the oncogenic pathways and their targets responsible for breast cancer pathogenesis. Thus studies on miRNAs would prove to be extremely beneficial not only to understand the underlying molecular mechanisms of metastasis but also for the advancement of diagnosis, prognosis, and treatment of cancer. Since specific miRNA signatures of patients help narrow down the stage of progression of cancer, it may effectively be used as non-invasive or minimally invasive cancer biomarkers and thus, enable researchers and medical practitioners to venture into a new realm of cancer therapy. These insights at molecular levels give impetus for identifying promising molecular biomarkers for early breast cancer diagnosis and prognosis in the future.

Numerous investigations revealed that these miRNAs are also found circulating in the blood system and could be easily quantified using q- RT-PCR [60]. This could lead to non-invasive liquid biopsy mode of diagnostic method that can track cancer in bodily fluids including blood, serum, plasma, or urine. This simple acquisition, detection, and stability of miRNAs in bodily fluids are critical and beneficial for their potential clinical uses [61, 62]. Exosomal miRNAs are also being used as non-invasive cancer markers because they have been found in all human physiological fluids, including plasma, serum, breast milk, saliva, bile, urine and cerebrospinal fluids [63]. Thus, molding the numerous miRNAs that have been shown to be dysregulated in our study into efficient diagnostic or prognostic biomarkers holds great promise for cancer clinical management.

## Conclusion

Due to lack of reliable markers for early diagnosis and prognosis, the discovery of cancer biomarkers has become a major focus of cancer research. Our study demonstrated the potential and prospective IDC-specific miRNAs to serve as active breast cancer biomarkers for scientific and clinical applications. We have identified and validated type, grade and stage specific markers for breast IDC. In silico analyses identified various oncogenic targets that were validated by Taqman individual assays, tissue microarrays, q-PCR, immunoblotting, immunocytochemistry and antibody array approaches. Though we present a repertoire of clinical data here, further extensive validation of individual miRNAs and clinical trials are needed to establish their role as early blood-based biomarkers in liquid biopsy systems for diagnosis and prognosis of IDC. Given the identified miRNAs’ potential as diagnostic, we propose a pathway for future clinical trials. If this could be further developed into a lab-on-chip, it might prove to be a boon for rural women in developing countries where women are reluctant to submit themselves for physical examination. Thus, a better understanding of the putative miRNA targets through in silico analysis, extensive validation, and massive clinical trials with an uncluttered approach would open up different standpoints for more refined, cost-effective, and non-invasive methods in breast cancer diagnosis. Our study paves the way for the clinical application of miRNAs as biomarkers in breast cancer by undertaking global screening of world population. The next steps involve rigorous validation of these miRNAs in larger patient cohorts, followed by the development and implementation of clinical trials to test their efficacy in the clinical practice.

### Supplementary Information

**Additional file 1: Supplementary Figure S1.** The morphological assessment of human breast cancer tissues of distinct grades was conducted. A. Illustrates a hyperchromatic nucleolus. B. Grade 1 IDC showcases numerous tubules, mild pleomorphic nuclei, and minimal mitotic activity. C. Demonstrates grade 2 IDC characterized by a reduced number of tubules, along with moderate pleomorphism and mitotic activity. D. Grade 3 reveals the absence of tubules, accompanied by high pleomorphism and mitotic activity. **Supplementary Figure S2.** Comparative analysis of common miRNA expression on TLDA and LNA array platform based on different grades, stages and estrogen receptors expression. A. Heat map expression of common miRNAs in TLDA and LNA Array across grade 2 and grade 3 along with their adjacent normal samples. Color scale shows 0 to -3 (Blue) log_10_ RQ (low expression) while 0 to +3 (Red) log_10_ RQ (high expression). B. Heat map shows the common miRNAs expression signature in ER+ve, ER-ve, Grade 2 (GR2), Grade 3(GR3), Grade 2 Stage I (GR2-Stg1), Grade 2 Stage II (GR2-Stg1I), Grade 2 Stage III (GR2-Stg1II), Grade 3 Stage I (GR3-Stg1), Grade 3 Stage II (GR3-Stg1I), Grade 3 Stage III (GR3-Stg1II). Color scale shows 0 to -3 (red) log10 RQ (low expression) while 0 to +3(green) log_10_ RQ (high expression). **Supplementary Figure S3.** Correlation of specific miRNAs clusters in grade 2 and grade 3 of human breast cancer. Correlation coefficient (R^2^) of miRNA cluster miR-19b-20a in grade 2 and grade 3 (0.971 & 0.971), let-7-c-99a in grade 2 and grade 3 (0.862 &0.955), miR-19a-19b in grade 2 and grade 3 (0.942 & 0.970), miR-145-2143 in grade 2 and grade 3 (0.812 & 0.912) all indicating a strong positive correlation among the subsets. Absolute correlation coefficient (R^2^) is equal to 1. **Supplementary Figure S4.** Individual assay validation of highly significant and common miRNAs. A. List of highly significant (p-values < 0.005) miRNAs up/down regulated in each ER+ve, ER-ve, GR2, GR3, stage I, stage II and stage III for individual assay validation. B. Hsa-miR-190b, hsa-miR-224 and hsa-miR-452 in ER+ve and hsa-miR-126^*^, hsa-miR-9 and hsa-miR-137 in ER-ve were validate using individual taqman probes and compared with respective miRs TLDA expression. C. Grade 2 specific miRs like hsa-miR-26a-2^*^ and hsa-miR-129-3p, while Grade 3 hsa-miR-767-5p and hsa-miR-147b individual assay in comparison with TLDA. Individual miRNAs expression represented as green bar (individual assay) and compared with yellow bar (TLDA) from stage I, II and III of grade 2 and Grade 2. D. Individual miRNAs (miR-155, miR-182, miR-429, and miR-141) expression from Stage II and Stage III of grade 2 and grade 3 were performed using individual Taqman probes and compared with respective miRNAs TLDA. Log_10_RQ 1 is 10 fold of expression. **Supplementary Figure S5.** Significant miRNAs in Grade 2 and Grade 3: A. List of 18 significant (p values 0.002 to 9.09E-06) miRNAs in grade 2 and non-significant (p values 0.063 to 0.99) in grade 3. The expression is represented by heat map with the scale (-3 to +3) where pink colour shows up regulation and red down regulation. The respective miRNAs (red dots) and their specific targets (blue dots) interaction are displayed using clustal analysis tool. C. Grade 3 ten significant (p values 0.01 to 8.52E-07) miRNAs vs non-significant (p values 0.1 to 0.97) in grade 3 along with heat map representation of expression pattern with the scale (-3 to +3) where green shows up regulation and red shows down regulation. miRNA (red node)-target (blue node) interaction analysis by clustal tool using R –program. **Supplementary Figure S6.** Significant miRNAs in stage I, II and III of Grade 2: A. Stage I significant miRNAs (6) with p values between 0.003 to 0.006 and non-significant stage II and III with p values between 0.092 to 0.99). The expression is represented by heat map with the scale (-3 to +3) where green colour shows up regulation and red down regulation. The respective miRNAs (red dots) and their specific targets (blue dots) interaction are displayed using cluster analysis tool. B. Stage II nineteen significant (p values 0.01 to 0.0005) miRNAs vs non-significant ( p values 0.1 to 0.97) in stage I and Stage III along with heat map representation of expression pattern with the scale (-3 to +3) where pink shows up regulation and red shows down regulation. The respective miRNA (red node)-target (blue node) interaction analysis by clustal tool. C. Stage III eight significant (p values 0.01 to 0.0003) miRNAs vs non-significant ( p values 0.1 to 0.97) in stage I and Stage II along with heat map representation of expression pattern with the scale (-3 to +3) where green shows up regulation and red shows down regulation. The respective miRNA (red node)-target (blue node) interaction analysis by clustal tool using R –program. **Supplementary Figure S7.** Significant miRNAs in stage I, II and III of Grade 3: A. Stage I significant miRNAs (18) with p values between 0.001 to 6.98E-05 and non-significant stage II and III with p values between 0.1 to 0.97). The expression is represented by heat map with the scale (-3 to +3) where green colour shows up regulation and red down regulation. The respective miRNAs (red dots) and their specific targets (blue dots) interaction are displayed using clustal analysis tool, B. Stage II six significant (p values 0.003 to 0.0008) miRNAs vs non-significant (p values 0.061 to 0.59) in stage I and stage III along with heat map representation of expression pattern with the scale (-3 to +3) where green shows up regulation and red shows down regulation. The respective miRNA (red node)-target (blue node) interaction analysis by clustal tool. C. Stage III four significant (p values 0.001 to 0.0008) miRNAs vs non-significant ( p values 0.1 to 0.99) in stage I and stage II along with heat map representation of expression pattern with the scale (-3 to +3) where green shows up regulation and red shows down regulation. The respective miRNA (red node)-target (blue node) interaction analysis by clustal tool using R –program. **Supplementary Figure S8.** Significant miRNAs in ER+ve and ER-ve, A. List of 17 significant (p values 0.0003 to 2.4E-14) miRNAs in ER+ve and non-significant (p values 0.12 to 0.98) in ER-ve. The expression is represented by heat map with the scale (-3 to +3) where green colour shows up regulation and red down regulation. The respective miRNAs (red dots) and their specific targets (blue dots) interaction are displayed using cluster analysis tool. B. ER-ve, nineteen significant (p values 0.01 to 2.59E-05) miRNAs vs non-significant ( p values 0.1 to 0.97) in ER+ve along with heat map representation of expression pattern with the scale (-3 to +3) where green shows up regulation and red shows down regulation. miRNA (red node)-target (blue node) interaction analysis by clustal tool using R –program. **Supplementary Figure S9.** MicroRNA-target interaction network and pathway enrichment analysis by GeneGO Metacore web computational tool. Highly significant and individually validated miRNAs were analysed for its specific targets involving in various oncogenic pathways A. MAPKinase, B. Notch signaling, C. VEGF signaling and D. Wnt signaling. Different coloured connecting lines between miRNAs and targets shows gene association, gene interaction, coexistence and expressions. Different coloured shape nodes represents enzymes transcription factors, receptors, ligands etc., where miRNA are represented as spiral single strand nucleotides. **Supplementary Figure S10.** In vitro validation of putative targets of significant miRNAs using miRNA mimics in MDA-MB 231 cells. Transfection of miRNA mimics (miR-432, miR-659, miR-105, miR-921, miR-662 and miR-21, scrambled as a control) and immunofluorescence of targets A. p53, B. p-β-catenin and C. APC. DAPI used to stain the nucleus, Phycoerythrin (PE-543) labelled with secondary antibody. The confocal image (DAPI channel, PE channel and Merged) was captured at 63X objective at 10μm scale. **Supplementary Figure S11.** In vitro validation of putative targets of significant miRNAs using miRNA mimics in MCF7 cells. Transfection of miRNA mimics (miR-921, miR-105, miR-432, miR-662 miR-659, miR-200c and miR-21, scrambled as a control) and immunoblotting for the targets A. p53, B.APC, PTEN, CASP3 and c-Myc C. PTEN, and D. BCL2. Beta-actin used as endogenous control and were used to normalize the target expression in densitometry plots. Band intensity was measured in triplicate and plotted with error bars. **Supplementary Figure S12.** Significant miRNAs from grade 2 (Stage I & II) and grade 3 (Stage I & II) target validation using antibody array. Antibody array of multiple targets of A. grade 2 stage I, B. grade 2 stage III, C. grade 3 Stage I, and D. grade 3 stage III. Protein samples from adjacent tissue (non cancerous) were used as normal control. Color range depicts the up regulation (+0.4 red color) and down regulation (-0.4 blue color). The yellow color indicates no expression.

**Additional file 2.**

## Data Availability

The complete data generated from this study is available at Gene Expression Omnibus GSE144463 (for TLDA) and GSE60725 (for LNA arrays) at http://www.ncbi.nlm.nih.gov/geo/. All the protocols associated with this study can be made available by email request to the corresponding author.

## Abbreviations

APC: Adenomatous polyposis coli
Bcl-2: B-cell lymphoma 2
BRCA1/Brca2: Breast cancer gene1/ 2
CASP3: Caspase 3
CASP8: Caspase 8
cDNA: Complementary DNA
c-Myc: cellular myelocytomatosis
ER +: Estrogen receptor positive
ISH: In situ Hybridization
KD: Knock Down
LCMS: Liquid Chromatography Mass Spectrometry
LNA: Locked nucleic acids
MDM2: Murine Double Minute 2
miRNA: micro RNA
OncomiRs: Oncogenic miRNAs
p53: Tumor protein P53
PAX5: Paired Box 5
PGR: Progesterone Receptor
PR +: Progesterone receptor positive
PTEN: Phosphatase and Tensin Homolog
qRT-PCR: quantitative Real-Time PCR
RCC: Regional Cancer Centre
RIN: RNA integrity number
RNA: Ribonucleic acid
STAT3: Signal Transducer And Activator Of Transcription 3
TLDA: TaqMan Low Density array
Wnt: Wingless-related integration site
