# Supplementary material for: MicroRNA signatures differentiate types, grades, and stages of breast invasive ductal carcinoma (IDC): miRNA-target interacting signaling pathways
Source: Cell Commun Signal. 2024 Feb 7;22:100. doi: 10.1186/s12964-023-01452-2 (PMC10851529; doi:10.1186/s12964-023-01452-2)
Supplement: Supplementary file 1 — Additional file 1: Supplementary Figure S1. The morphological assessment of human breast cancer tissues of distinct grades was conducted. A. Illustrates a hyperchromatic nucleolus. B. Grade 1 IDC showcases numerous tubules, mild pleomorphic nuclei, and minimal mitotic activity. C. Demonstrates grade 2 IDC characterized by a reduced number of tubules, along with moderate pleomorphism and mitotic activity. D. Grade 3 reveals the absence of tubules, accompanied by high pleomorphism and mitotic activity. Supplementary Figure S2. Comparative analysis of common miRNA expression on TLDA and LNA array platform based on different grades, stages and estrogen receptors expression. A. Heat map expression of common miRNAs in TLDA and LNA Array across grade 2 and grade 3 along with their adjacent normal samples. Color scale shows 0 to -3 (Blue) log10 RQ (low expression) while 0 to +3 (Red) log10 RQ (high expression). B. Heat map shows the common miRNAs expression signature in ER+ve, ER-ve, Grade 2 (GR2), Grade 3(GR3), Grade 2 Stage I (GR2-Stg1), Grade 2 Stage II (GR2-Stg1I), Grade 2 Stage III (GR2-Stg1II), Grade 3 Stage I (GR3-Stg1), Grade 3 Stage II (GR3-Stg1I), Grade 3 Stage III (GR3-Stg1II). Color scale shows 0 to -3 (red) log10 RQ (low expression) while 0 to +3(green) log10 RQ (high expression). Supplementary Figure S3. Correlation of specific miRNAs clusters in grade 2 and grade 3 of human breast cancer. Correlation coefficient (R2) of miRNA cluster miR-19b-20a in grade 2 and grade 3 (0.971 & 0.971), let-7-c-99a in grade 2 and grade 3 (0.862 &0.955), miR-19a-19b in grade 2 and grade 3 (0.942 & 0.970), miR-145-2143 in grade 2 and grade 3 (0.812 & 0.912) all indicating a strong positive correlation among the subsets. Absolute correlation coefficient (R2) is equal to 1. Supplementary Figure S4. Individual assay validation of highly significant and common miRNAs. A. List of highly significant (p-values < 0.005) miRNAs up/down regulated in each ER+ve, ER-ve, GR2, GR3, stage I, st [file 12964_2023_1452_MOESM1_ESM.docx]

**Supplementary Figures**

**“MicroRNA signatures differentiate Types, Grades, and Stages of Breast Invasive Ductal Carcinoma (IDC): miRNA-target interacting signaling pathways”**

Vinod Kumar Verma^1^, ^δ^Syed Sultan Beevi^1^, ^δ^Rekha A Nair ^2^**^*^**, Aviral Kumar^1^ Ravi Kiran^1^, Liza Esther Alexander^2^, ^*^Lekha Dinesh Kumar^1^

^1^Cancer Biology, CSIR-Centre for Cellular and Molecular Biology, (CCMB) Uppal Road, Hyderabad, 500007, Telangana, India.

^2^Department of Pathology and Medical Oncology, Regional Cancer Centre (RCC), Medical College Campus, Trivandrum, 695011, India

^δ^These authors contributed equally to this work

^*^Corresponding author: **Dr. Lekha Dinesh Kumar**, Sr. Principal Scientist, Project Leader, Cancer Biology, CSIR-CCMB, Hyderabad, Email id- [lekha@ccmb.res.in](mailto:lekha@ccmb.res.in)


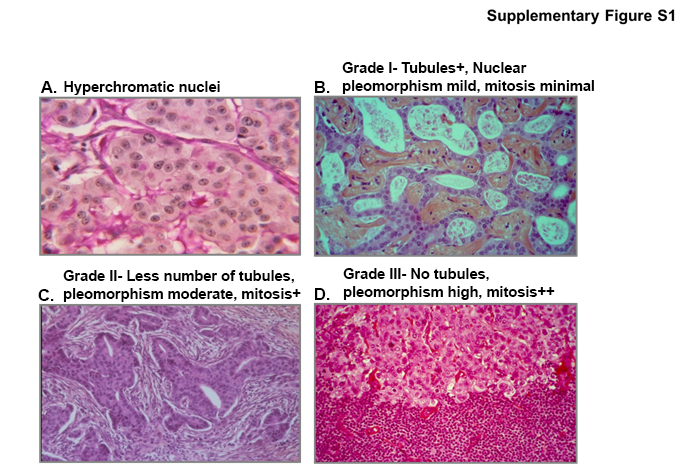


**Supplementary Figure S1: The morphological assessment of human breast cancer tissues of distinct grades**. **A**. Illustrates hyperchromatic nuclei. **B.** Grade 1 IDC showcases numerous tubules, mild pleomorphic nuclei, and minimal mitotic activity. **C.** Grade 2 IDC sample characterized by a reduced number of tubules, along with moderate pleomorphism and mitotic activity. **D.** Grade 3 reveals the absence of tubules, accompanied by high pleomorphism and increased mitotic activity.


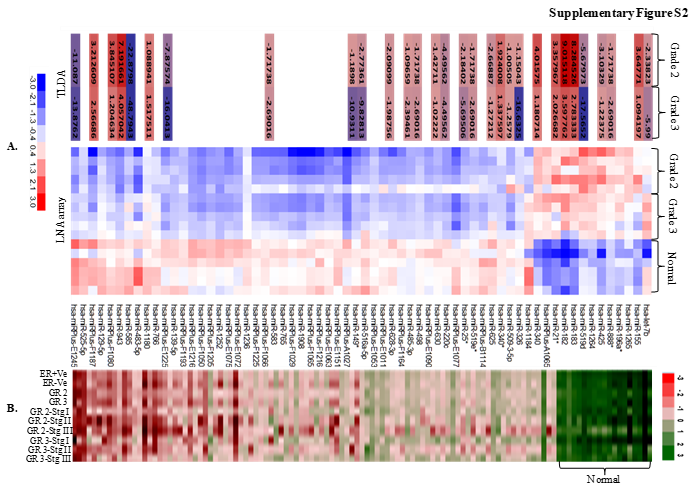


**Supplementary Figure S2: Comparative analysis of common miRNA expression on TLDA and LNA platform based on different grades, stages and estrogen receptor expression**. **A.** Heat map expression of common miRNAs in TLDA and LNA arrays across grade 2 and grade 3 along with their adjacent normal samples. Color scale shows 0 to -3 (Blue) log_10_ RQ (low expression) while 0 to +3 (Red) log_10_ RQ (high expression). **B.** Heat map shows the common miRNAs expression signature in ER+ve, ER-ve, Grade 2 (GR2), Grade 3(GR3), Grade 2 Stage I (GR2-Stg1), Grade 2 Stage II (GR2-Stg1I), Grade 2 Stage III (GR2-Stg1II), Grade 3 Stage I (GR3-Stg1), Grade 3 Stage II (GR3-Stg1I), Grade 3 Stage III (GR3-Stg1II). Color scale shows 0 to -3 (red) log10 RQ (low expression) while 0 to +3(green) log_10_ RQ (high expression).


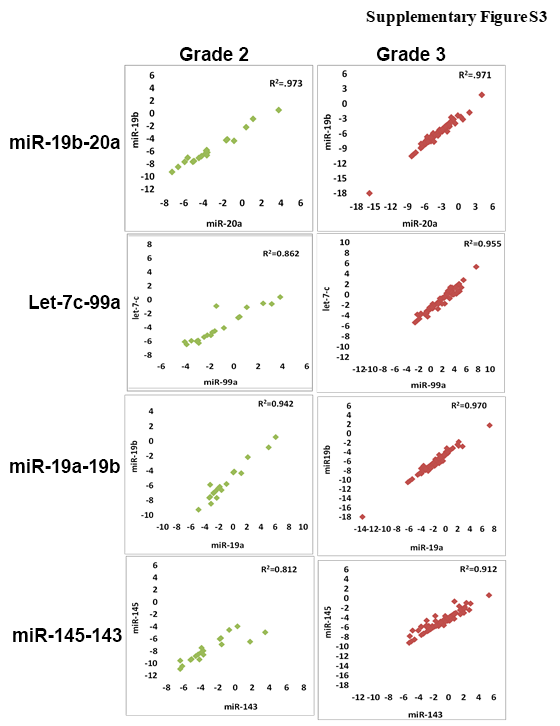


**C**

**B**

**A**

**D**

**Supplementary Figure S3: Correlation of specific miRNAs clusters in grade 2 and grade 3 of IDC samples.** Correlation coefficient (R^2^) of miRNA clusters **A.** miR-19b-20a in grade 2 and grade 3 (0.973 & 0.971), **B.** let-7-c-99a in grade 2 and grade 3 (0.862 &0.955), **C.** miR-19a-19b in grade 2 and grade 3 (0.942 & 0.970), **D.** miR-145-143 in grade 2 and grade 3 (0.812 & 0.912), all indicating a strong positive correlation among the subsets. Absolute correlation coefficient (R^2^) is equal to 1.

**
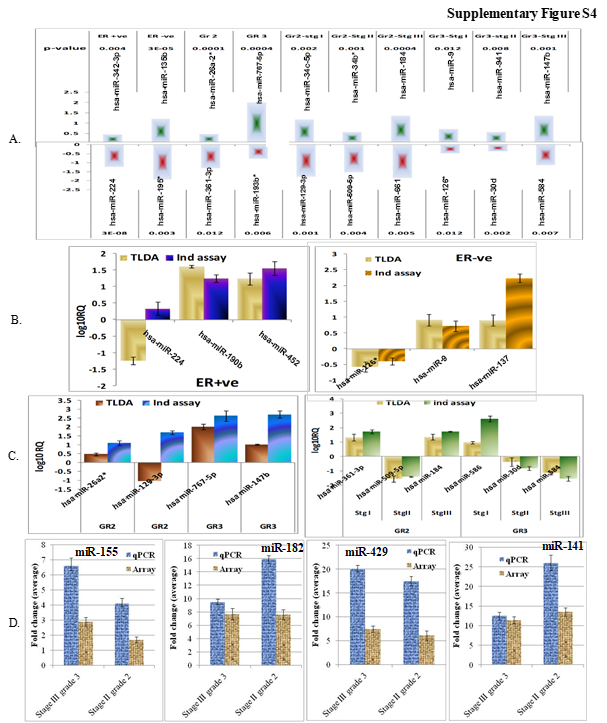
**

**Supplementary Figure S4: Taqman Individual assay validation of highly significant and common miRNAs. A**. List of highly significant (p-values < 0.005) miRNAs up/down regulated in ER+ve, ER-ve, GR2, GR3,GR2 stage I, Stage II and stage III for individual assay validation. **B.** Hsa- miR-224, hsa­­­­­-miR-190b, and hsa-miR-452 in ER+ve and hsa-miR-126^*^, hsa-miR-9 and hsa-miR-137 in ER-ve were validated using individual taqman probes. **C.** Grade 2 specific miRs like hsa-miR-26a2^*^ and has-miR-129-3p, while Grade 3 has-miR-767-5p and has-miR-147b. Stage I, II and III of grade 2 and Grade 3 respectively. **D.** Individual miRNAs (miR-155, miR-182, miR-429, and miR-141) expression from Stage II and Stage III of grade 2 and grade 3 were performed using individual Taqman probes and compared with respective miRNAs TLDA. Log_10_RQ 1 is 10 fold of expression.


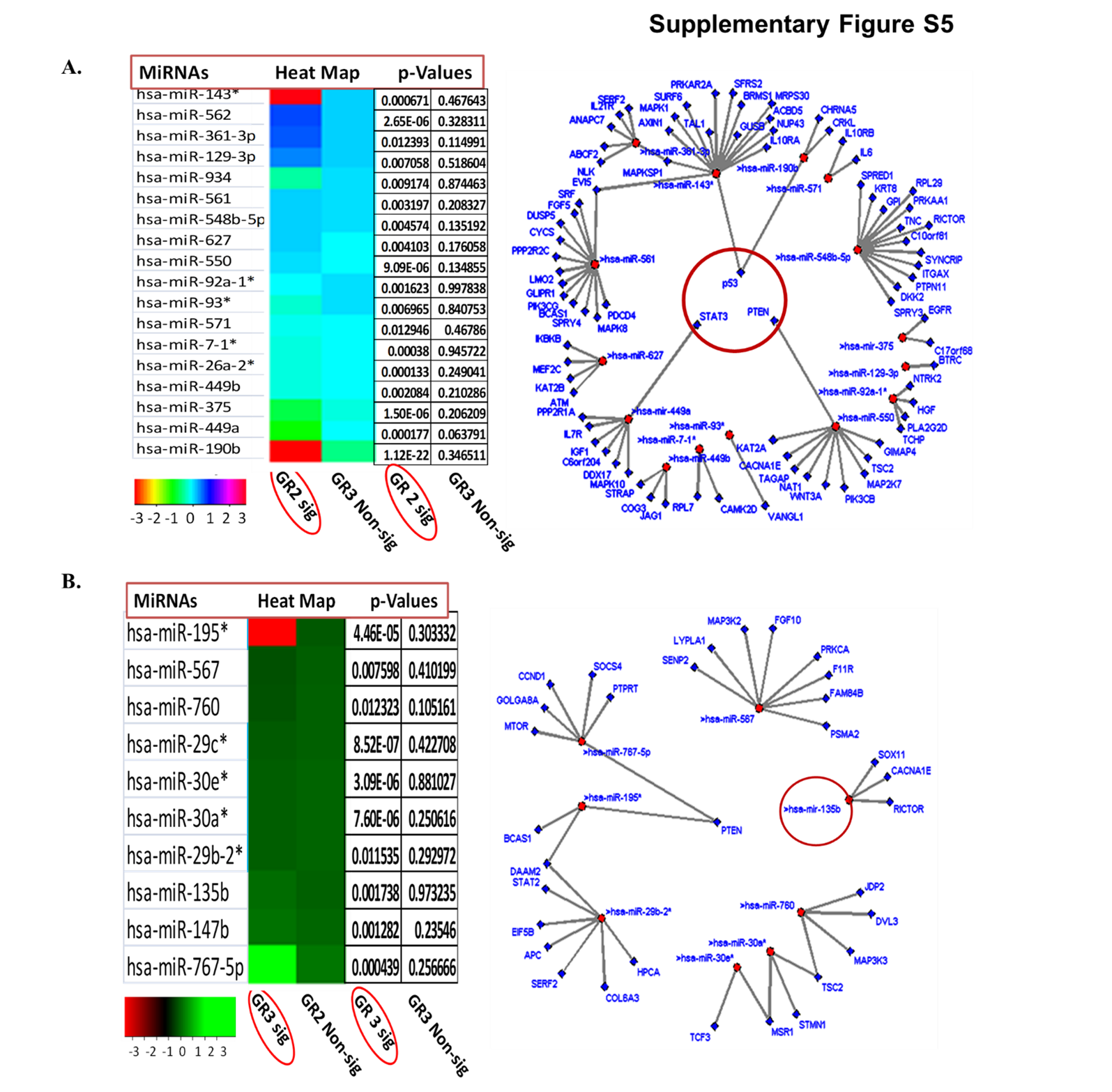


**Supplementary Figure S5**: **Significant miRNAs in Grade 2 and Grade 3**: **A.** List of 18 significant (p-values 0.002 to 9.09E-06) miRNAs in grade 2 and non-significant (p values 0.063 to 0.99) in grade 3. The expression is represented by heat map with the scale (-3 to +3) where pink colour shows up regulation and red indicates down regulation. The respective miRNAs (red nodes) and their specific targets (blue nodes) interaction are displayed using clustal analysis tool. **B.** Grade 3 ten significant (p values 0.01 to 8.52 E-07) miRNAs vs non-significant (p values 0.1 to 0.97) in grade 3 along with heat map representation of expression pattern with the scale (-3 to +3) where green shows up regulation and red shows down regulation. miRNA (red nodes) and target (blue nodes) interaction analysis by clustal tool using R –program.


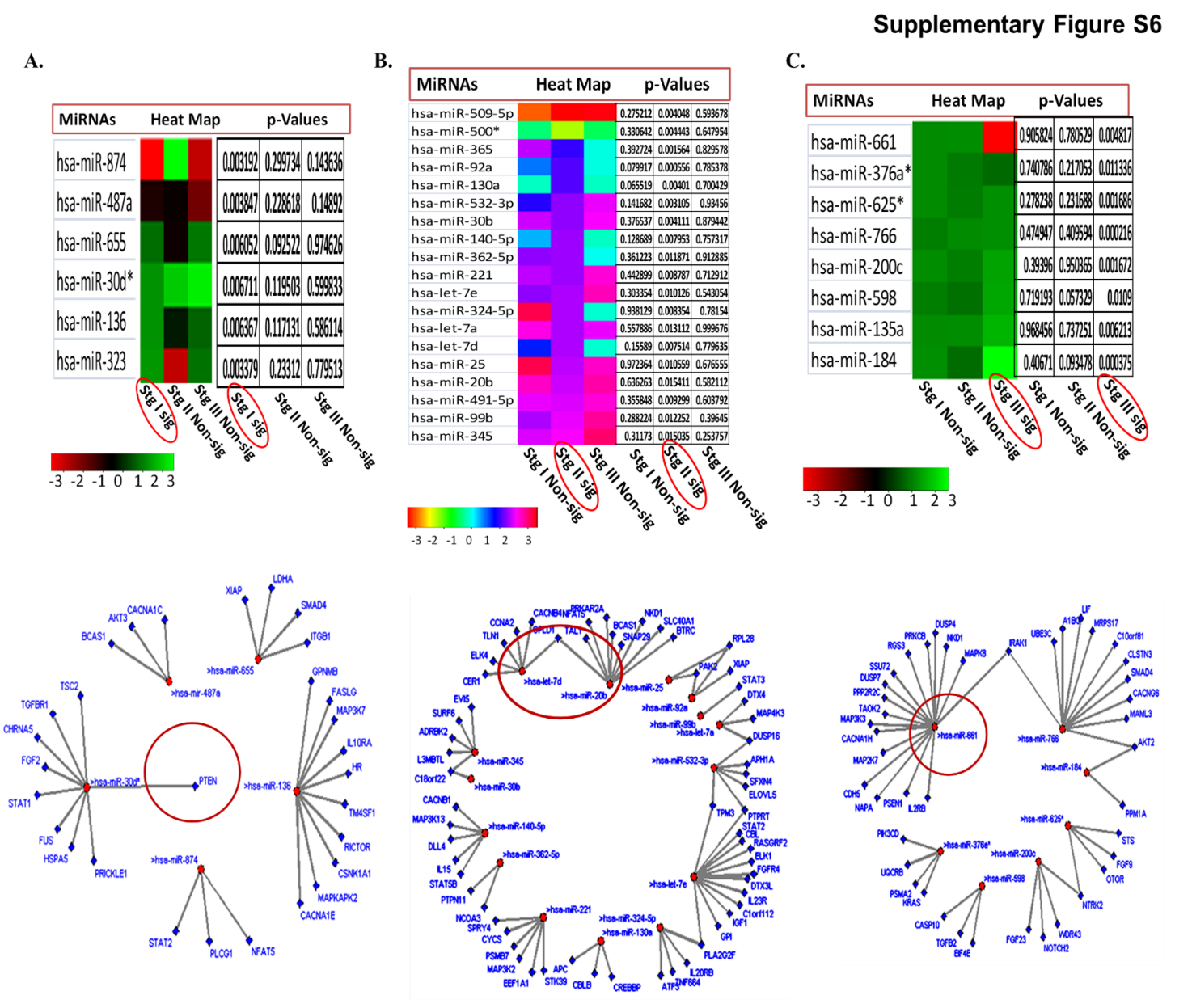


**Supplementary Figure S6**: **Significant miRNAs in stages I, II and III of Grade 2**: **A**. Stage I significant six miRNAs with p-values between 0.003 to 0.007 and non-significant stage II and III with p-values between 0.092 to 0.99). The expression is represented by heat map with the scale (-3 to +3) where green colour shows up regulation and red down regulation. The respective miRNAs (red nodes) and their specific targets (blue nodes) interaction are displayed using clustal analysis tool. **B.** Stage II nineteen significant (p-values 0.0005 to 0.02) miRNAs vs non-significant (p-values 0.5 to 1.00) in stage I and Stage III along with heat map representation of expression pattern with the scale (-3 to +3) where pink shows up regulation and red shows down regulation. The respective miRNA (red node) -target (blue node) interaction analysis by clustal tool. **C.** Stage III eight significant (p values 0.0001 to 0.02) miRNAs vs non-significant (p-values 0.05 to 1.00) in stage I and Stage II along with heat map representation of expression pattern with the scale (-3 to +3) where green shows up regulation and red shows down regulation. The respective miRNA (red node) and target (blue node) interaction analysis by clustal tool using R –program.


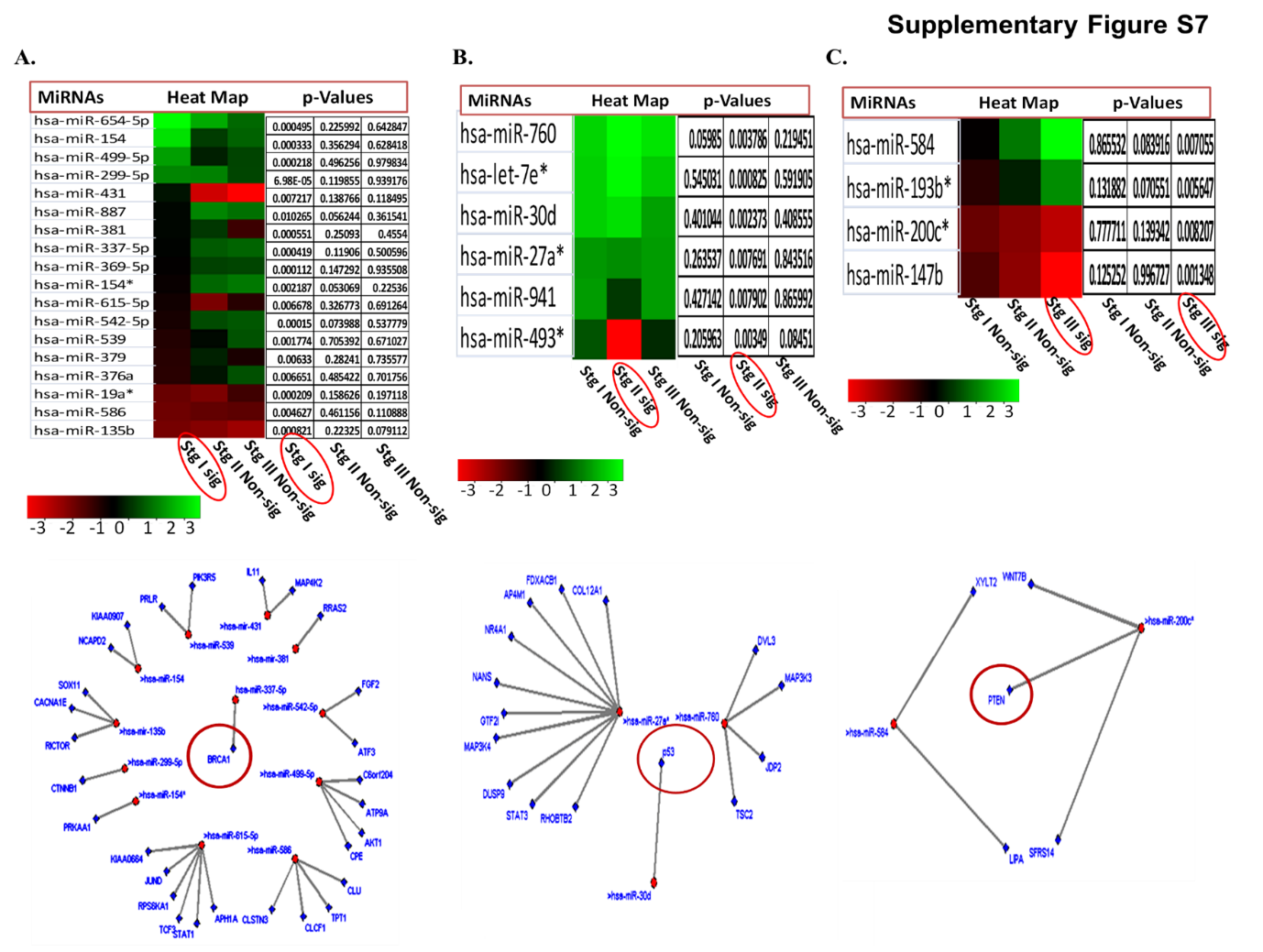


**Supplementary Figure S7**: **Significant miRNAs in stage I, II and III of Grade 3**: **A**. Eighteen stage I significant miRNAs with p-values (6.98E-05 to 0.02) and non-significant stage II and III with p-values between 0.052 to 0.98). The expression is represented by heat map with the scale (-3 to +3) where green colour shows up regulation and red down regulation. The respective miRNAs (Red nodes) and their specific targets (Blue nodes) interaction are displayed using clustal analysis tool, **B.** Stage II six significant (p-values 0.0008 to 0.90) miRNAs vs non-significant (p-values 0.05 to 0.60) in stage I and stage III along with heat map representation of expression pattern with the scale (-3 to +3) where green shows up regulation and red shows down regulation. The respective miRNA (red node)-target (blue node) interaction analysis by clustal tool, **C.** Stage III four significant (p values 0.001 to 0.009) miRNAs vs non-significant (p-values 0.07 to 0.10) in stage I and stage II along with heat map representation of expression pattern with the scale (-3 to +3) where green shows up regulation and red shows down regulation. The respective miRNA (red node)-target (blue node) interaction analysis by clustal tool using R –program.


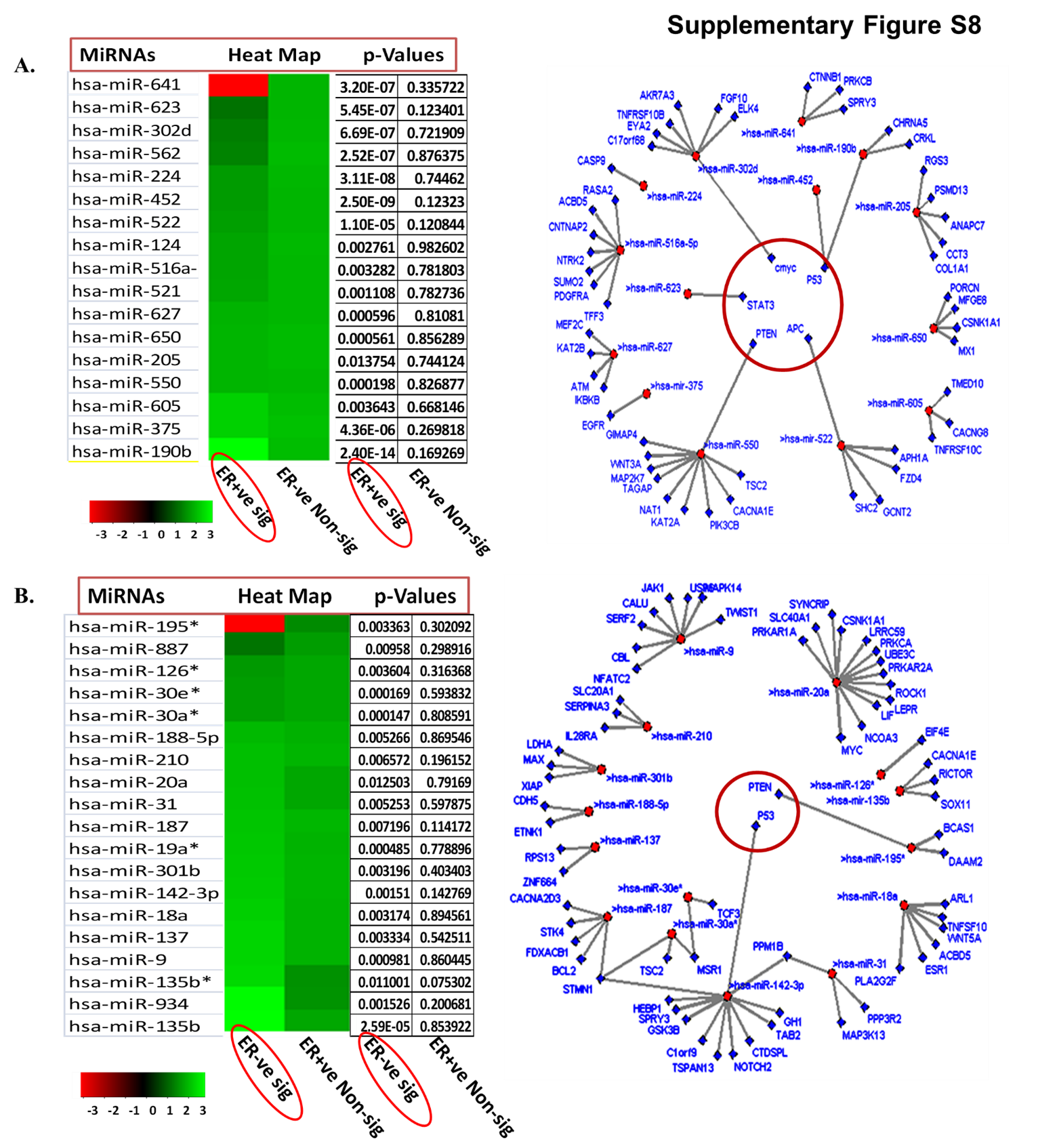


**B**.

**Supplementary Figure S8**: **Significant miRNAs in ER+ve ER-ve** **A**. List of 17 significant (p- values 2.4E-14 to 0.02) miRNAs in ER+ve and non-significant (p-values 0.12 to 1.00) in ER-ve. The expression is represented by heat map with the scale (-3 to +3) where green colour shows up regulation and red down regulation. The respective miRNAs (Red nodes) and their specific targets (Blue nodes) interaction are displayed using cluster analysis tool. **B.** ER-ve, nineteen significant (p- values 2.59E-05-0.02) miRNAs vs non-significant (p-values 0.07 to 0.97) in ER+ve along with heat map representation of expression pattern with the scale (-3 to +3) where green shows up regulation and red shows down regulation. miRNA (red node)-target (blue node) interaction analysis by clustal tool using R –program.

**D**.

**C**.


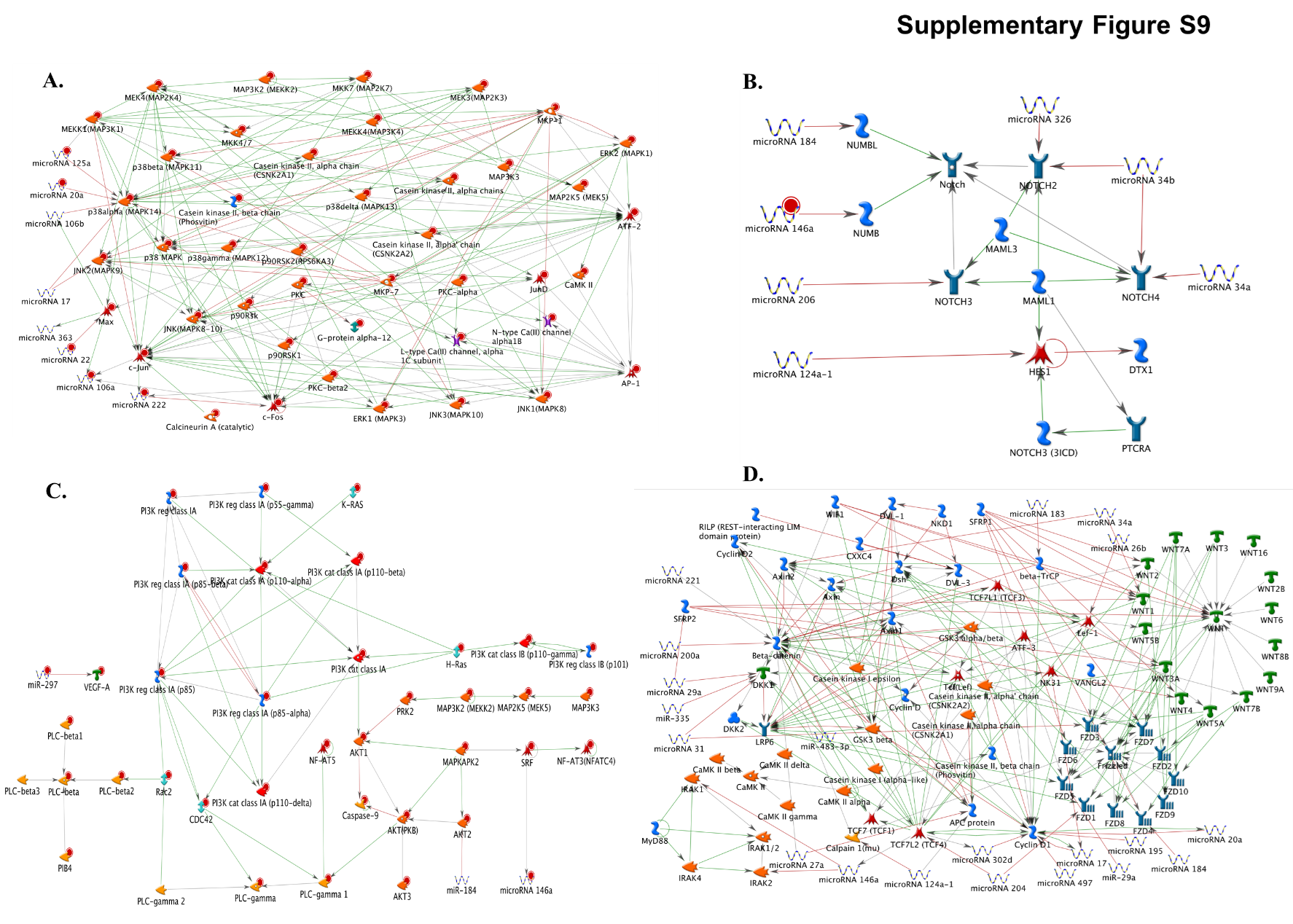


Wnt

VEGF

Notch

MAPK

**Supplementary Figure S9: MicroRNA-target interaction network and pathway enrichment analysis by GeneGO Metacore web computational tool**. Highly significant and individually validated miRNAs were analysed for its specific targets involved in various oncogenic pathways **A.** MAPKinase, **B.** Notch signaling, **C.** VEGF signaling and **D.** Wnt signaling. Different coloured connecting lines between miRNAs and targets shows gene association, gene interaction, coexistence and expressions. Different coloured shape nodes represents enzymes transcription factors, receptors, ligands etc., where miRNA are represented as spiral single strand nucleotides.


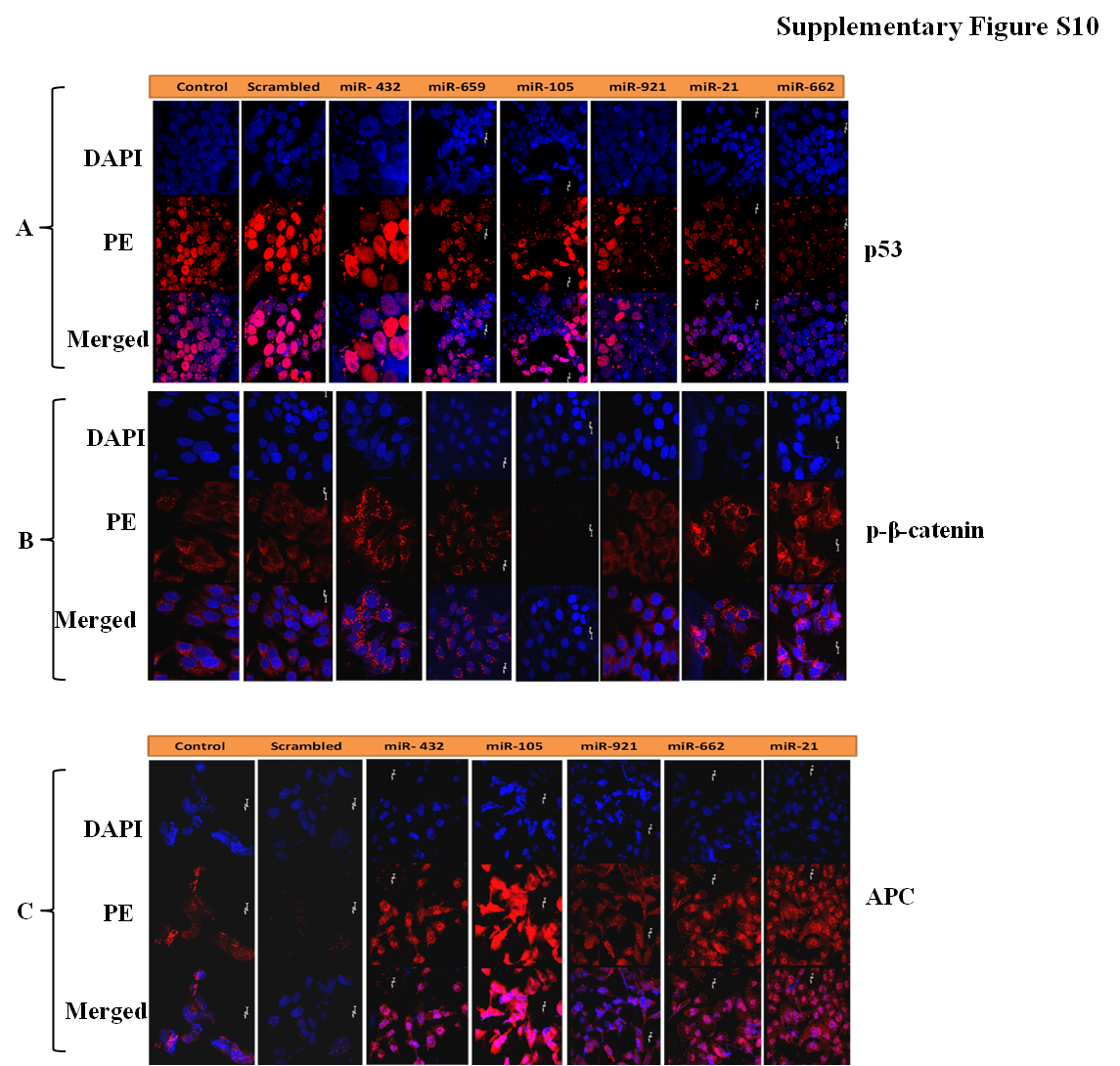


**Supplementary Figure S10**: **In vitro validation of putative targets of significant miRNAs using miRNA mimics in MDA-MB 231 cells**. Transfection of miRNA mimics (miR-432, miR-659, miR-105, miR-921, miR-21and miR-662 and scrambled as a control) and immunofluorescence of targets **A.** p53, **B**. p-β-catenin and **C**. APC. DAPI used to stain the nucleus, Phycoerythrin (PE-543) labelled with secondary antibody. The confocal image (DAPI channel, PE channel and Merged) was captured at 63X objective at 10µm scale.


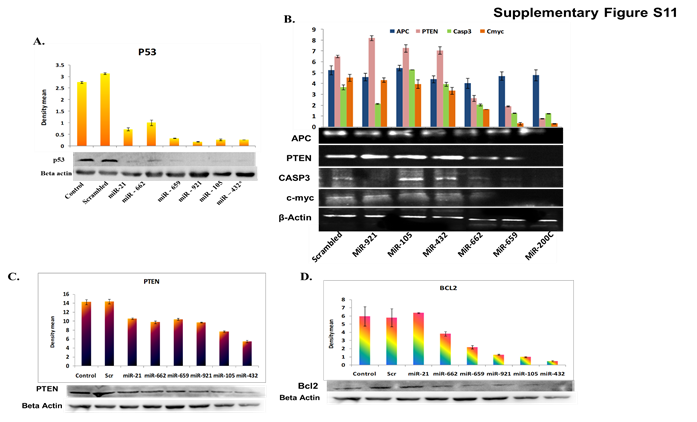


**Supplementary Figure S11: In vitro validation of putative targets of significant miRNAs using miRNA mimics in MCF7 cells.** Transfection of miRNA mimics (miR-921, miR-105, miR-432, miR-662 miR-659, miR-200c and miR-21, scrambled as a control) and immunoblotting for the targets **A.** p53, **B**.APC, PTEN, CASP3 and c-Myc **C**. PTEN and **D.** BCL2. β-actin used as endogenous control and were used to normalize the target expression in densitometry plots. Band intensity was measured in triplicate and plotted with error bars.


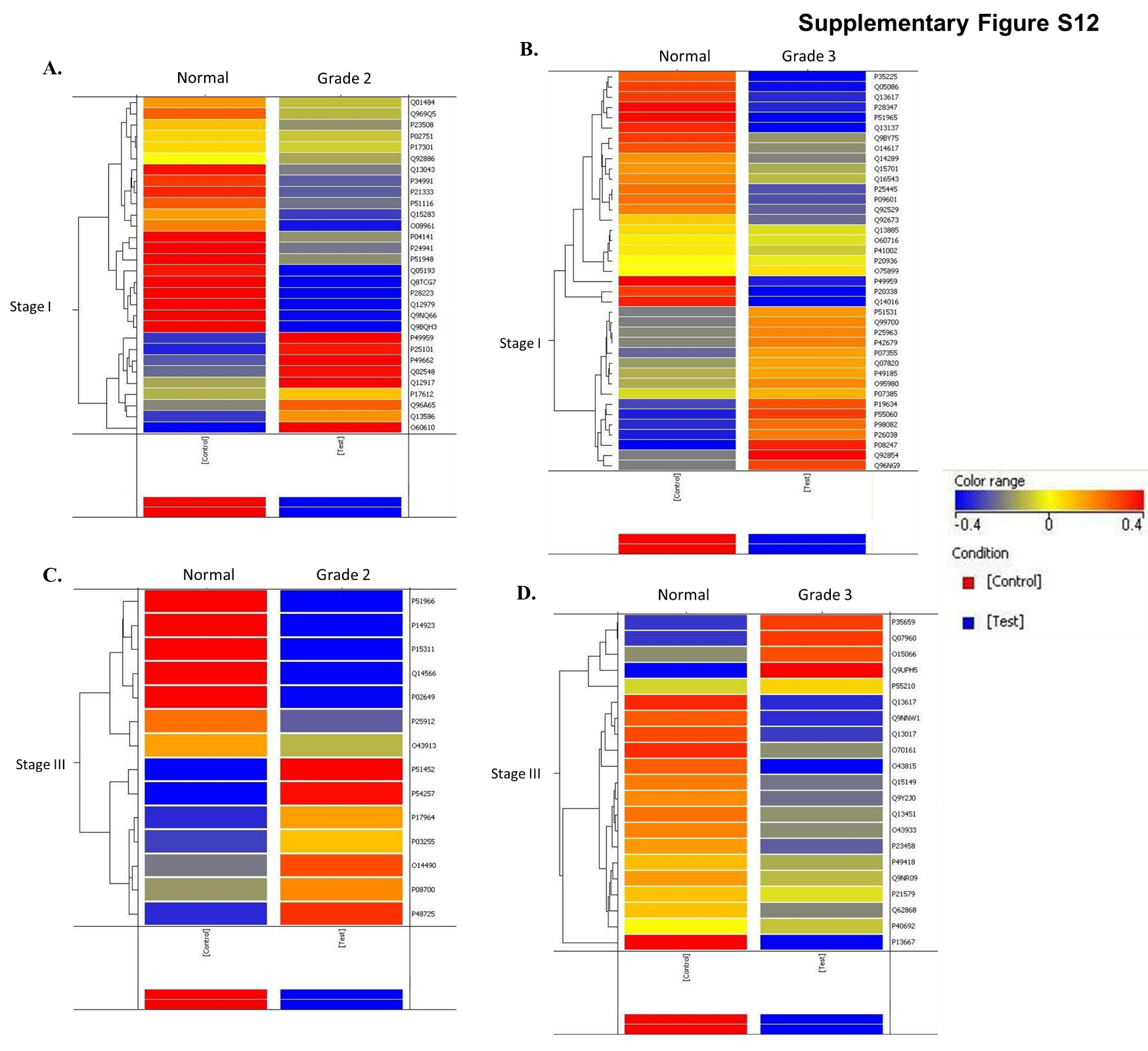


**Supplementary Figure S12:** Target validation using antibody array of significant miRNAs from Grade 2 (Stage I & II) and grade 3 (Stage I & II). Antibody array of multiple targets of **A.** grade 2 stage I, B. grade 3 stage I, **C.** Grade 2 Stage III and **D.** Grade 3 stage III. Protein samples from adjacent normal tissue were used as control. Color range depicts the up regulation (+0.4 red color) and down regulation (-0.4 blue color). The yellow color indicates no expression.
