# Supplementary material for: MicroRNA signatures differentiate types, grades, and stages of breast invasive ductal carcinoma (IDC): miRNA-target interacting signaling pathways
Source: Cell Commun Signal. 2024 Feb 7;22:100. doi: 10.1186/s12964-023-01452-2 (PMC10851529; doi:10.1186/s12964-023-01452-2)
Supplement: Supplementary file 2 — Additional file 2. [file 12964_2023_1452_MOESM2_ESM.pdf]

Supplementary Tables

"MicroRNA signatures differentiate Types, Grades, and Stages of Breast Invasive Ductal Carcinoma (IDC): miRNA-target interacting signaling pathways"

Vinod Kumar Verma[1], [δ]Syed Sultan Beevi[1], [δ]Rekha A Nair [2*], Aviral Kumar[1], Ravi Kiran, [1] Liza Esther Alexander[2], [*]Lekha Dinesh Kumar[1]

[1]Cancer Biology, CSIR-Centre for Cellular and Molecular Biology, (CCMB) Uppal Road, Hyderabad, 500007, Telangana, India.

[2]Department of Pathology and Medical Oncology, Regional Cancer Centre (RCC), Medical College Campus, Trivandrum, 695011, India

[δ]These authors contributed equally to this work

[*]Corresponding author: **Dr. Lekha Dinesh Kumar**, Sr. Principal Scientist, Project Leader, Cancer Biology, CSIR-CCMB, Hyderabad, Email id- lekha@ccmb.res.in

Supplementary Tables

Table S1: Pathway analysis of selected miRNAs with their putative targets and fold changes in Grade 2 and Grade 3

	Angiogenesis pathway									
	GradeII	fold change	fold change	fold change	Grade III	fold change	fold change	fold change		
	miRNAs	stageI	stageII	stageIII	miRNAs	stageI	stageII	stageIII	Target gene ID	
	hsa-miR-141	1.154842968	0.537407279	1.188771479	hsa-miR-141	0.72709	0.71856	1.49519	2045	EPH receptor A7
	hsa-miR-200a	1.110963857	0.255986663	1.063765537	hsa-miR-200a	0.63367	0.34486	1.15097	2045	EPH receptor A7
-1000	hsa-miR-204	-1.318326985	-1.322233061	-1.155651136	hsa-miR-204	-1.01404	-1.38216	-1.2376	2048	EPH receptor B2
1000	hsa-miR-369-3p	-1.085912231	-1.106741487	-0.937233268	hsa-miR-369-3p	-1.20286	-0.57231	-0.327	2247	fibroblast growth factor 2 (basic)
	hsa-miR-515-5p	-2.273928238	-2.055893452	-1.858580363	hsa-miR-515-5p	-1.51184	-1.25231	-0.8716	2263	fibroblast growth factor receptor 2
	hsa-miR-519c-3p	-2.169073831	-1.399534973	-0.677167073	hsa-miR-519c-3p	-1.24783	-0.53828	-1.3493	3091, 2043	hypoxia inducible factor 1, alpha subunit (basic helix-loop-helix transcription factor)
	hsa-miR-519d	-1.198935479	-1.526543203	-1.044663513	hsa-miR-519d	-0.76215	-0.94638	-1.026	3091, 57144, 2043, 3716	hypoxia inducible factor 1, alpha subunit (basic helix-loop-helix transcription factor)
	hsa-miR-520b	-1.605358484	-1.22887204	-1.200293553	hsa-miR-520b	-1.03131	-1.11905	-0.621	57144	p21 protein (Cdc42/Rac)-activated kinase 7
	hsa-miR-520g	-2.136470913	-2.461675719	-1.985960604	hsa-miR-520g	-1.55894	-1.3233	-1.1348	3091, 3716	hypoxia inducible factor 1, alpha subunit (basic helix-loop-helix transcription factor)
	hsa-miR-96-	1.067504036	0.17229519	1.15315224	hsa-miR-96	0.57186	1.20598	1.4138	10818	fibroblast growth factor receptor substrate 2
	P53 pathway									
	hsa-miR-141	1.154842968	0.537407279	1.188771479	hsa-miR-141	0.72709	0.71856	1.49519	23411	sirtuin (silent mating type information regulation 2 homolog)
	hsa-miR-200a	1.110963857	0.255986663	1.063765537	hsa-miR-200a	0.63367	0.34486	1.15097	23411	sirtuin (silent mating type information regulation 2 homolog)
	hsa-miR-515-5p	-2.273928238	-2.055893452	-1.858580363	hsa-miR-515-5p	-1.51184	-1.25231	-0.8716	472, 6613	ataxia telangiectasia mutated
	hsa-miR-519d	-1.198935479	-1.526543203	-1.044663513	hsa-miR-519d	-0.76215	-0.94638	-1.026	1026	cyclin-dependent kinase inhibitor 1A (p21, Cip1)
	hsa-miR-520b	-1.605358484	-1.22887204	-1.200293553	hsa-miR-520b	-1.03131	-1.11905	-0.621	8850	K(lysine) acetyltransferase 2B
	T-Cell Activation									
	hsa-miR-141	1.154842968	0.537407279	1.188771479	hsa-miR-141	0.72709	0.71856	1.49519	941	CD80 molecule
	hsa-miR-200a	1.110963857	0.255986663	1.063765537	hsa-miR-200a	0.63367	0.34486	1.15097	941	CD80 molecule
	hsa-miR-520g	-2.136470913	-2.461675719	-1.985960604	hsa-miR-520g	-1.55894	-1.3233	-1.1348	10451	vav 3 guanine nucleotide exchange factor
	Apoptosis pathway									
	hsa-miR-141	1.154842968	0.537407279	1.188771479	hsa-miR-200a	0.63367	0.34486	1.15097	6416	mitogen-activated protein kinase kinase 4
	hsa-miR-204	-1.318326985	-1.322233061	-1.155651136	hsa-miR-204	-1.01404	-1.38216	-1.2376	329	baculoviral IAP repeat-containing 2
	hsa-miR-369-3p	-1.085912231	-1.106741487	-0.937233268	hsa-miR-369-3p	-1.20286	-0.57231	-0.327	330	baculoviral IAP repeat-containing 3
	hsa-miR-429	1.112554304	0.323170433	1.265133879	hsa-miR-429	0.90132	0.71115	1.40957	11183, 1965	mitogen-activated protein kinase kinase kinase kinase 5
	TGB-beta sig pathway									
	hsa-miR-204	-1.318326985	-1.322233061	-1.155651136	hsa-miR-204	-1.01404	-1.38216	-1.2376	10890	RAB10, member RAS oncogene family
	hsa-miR-224	-1.292366399	-1.682497297	-1.008850607	hsa-miR-369-3p	-1.20286	-0.57231	-0.327	4089	SMAD family member 4
	hsa-miR-369-3p	-1.085912231	-1.106741487	-0.937233268	hsa-miR-515-5p	-1.51184	-1.25231	-0.8716	7093, 659	tolloid-like 2
	hsa-miR-515-5p	-2.273928238	-2.055893452	-1.858580363	hsa-miR-519c-3p	-1.24783	-0.53828	-1.3493	7048	transforming growth factor, beta receptor II
	hsa-miR-519c-3p	-2.169073831	-1.399534973	-0.677167073	hsa-miR-520b	-1.03131	-1.11905	-0.621	659, 7048	bone morphogenetic protein receptor, type II
	hsa-miR-520b	-1.605358484	-1.22887204	-1.200293553	hsa-miR-592	1.54767	1.40689	1.54855	7048, 25805	transforming growth factor, beta receptor II
	hsa-miR-592	1.683568289	1.746206352	1.811312618					92	activin A receptor, type IIA
	Wnt pathway									
	hsa-miR-204	-1.318326985	-1.322233061	-1.155651136	hsa-miR-204	-1.01404	-1.38216	-1.2376	6596	helicase-like transcription factor
	hsa-miR-369-3p	-1.085912231	-1.106741487	-0.937233268	hsa-miR-369-3p	-1.20286	-0.57231	-0.327	8323, 7091	frizzled homolog 6
	hsa-miR-422a	-1.1533268	-1.406072618	-0.547654456	hsa-miR-422a	-0.56842	-0.79568	-0.9197	1495	catenin (cadherin-associated protein), alpha 1
	hsa-miR-429	1.112554304	0.323170433	1.265133879	hsa-miR-452	-0.93717	-0.73653	-0.6498	51384	wingless-type MMTV integration site family, member 16
	hsa-miR-452	-1.384097193	-1.735993612	-1.053738424	hsa-miR-512-3p	-1.01764	-0.92036	-1.0393	5529	protein phosphatase 2, regulatory subunit B', epsilon isoform
	hsa-miR-512-3p	-1.233115463	-1.480787127	-1.011933019	hsa-miR-519c-3p	-1.24783	-0.53828	-1.3493	5529	protein phosphatase 2, regulatory subunit B', epsilon isoform
	hsa-miR-519c-3p	-2.169073831	-1.399534973	-0.677167073	hsa-miR-520g	-1.55894	-1.3233	-1.1348	23291	F-box and WD repeat domain containing 11
	hsa-miR-520g	-2.136470913	-2.461675719	-1.985960604	hsa-miR-96	0.57186	1.20598	1.4138	23291	F-box and WD repeat domain containing 11
	hsa-miR-96	1.067504036	0.17229519	1.15315224	hsa-miR-767-5p	2.27438	2.44531	1.46469	5534, 6907	protein phosphatase 3 (formerly 2B), regulatory subunit B, alpha isoform
	Ras pathway									
	hsa-miR-519d	-1.198935479	-1.526543203	-1.044663513	hsa-miR-519d	-0.76215	-0.94638	-1.026	23179	ral guanine nucleotide dissociation stimulator-like 1

Table S2: Protein array revealed differential expression of protein with their corresponding miRNAs & their oncogenic pathways in different grades and stages

Grade II Stage I

SP ID	Target name	Regulation	Fold Change	miRNA	Apoptosis	Cancer	Cell Cycle	Ptn Kinases	
P28223	5-hydroxytryptamine (serotonin) receptor 2A	down	↑ 21.934555	hsa-miR-105					
Q9NQ66	phospholipase C, beta 1 (phosphoinositide-specific)	down	→ 11.2731	hsa-miR-141		+			
P02751	fibronectin 1	down	→ 10.627422	hsa-miR-429		+			↑ -100
Q13043	serine/threonine kinase 4	down	↓ 4.927109	hsa-miR-183	+			+	
P51116	fragile X mental retardation, autosomal homolog 2	down	↓ 4.6091144	hsa-miR-182*					
Q01484	ankyrin 2, neuronal	down	↓ 1.9440357	hsa-miR-93			+		
Q12979	active BCR-related gene	down	↓ 1.5373068	hsa-miR-19a			+		↑ 100
P04141	colony stimulating factor 2 (granulocyte-macrophage	down	↓ 1.2546576	hsa-miR-34a		+			
P34991	S-phase kinase-associated protein 1A (p19A)	down	↓ 1.2266519	hsa-miR-130b		+	+		
P24941	cyclin-dependent kinase 2	down	↓ 1.1503014	hsa-miR-148b	+	+	+	+	
Q05193	dynamin 1	down	↑ -1.295874	hsa-miR-24	+	+			
Q969Q5	RAB24, member RAS oncogene family	down	→ -1.733576	hsa-miR-370		+			
P23508	mutated in colorectal cancers	down	↓ -2.469643	hsa-miR-214	+	+			
P49959	MRE11 meiotic recombination 11 homolog A (S. cere	up	→ -8.239151	hsa-miR-378			+		
P25101	endothelin receptor type A	up	↑ -2.469643	hsa-miR-214					
P49662	caspase 4, apoptosis-related cysteine protease	up	→ -8.239151	hsa-miR-378	+		+		
P17612	protein kinase, cAMP-dependent, catalytic, alpha	up	↓ -12.7369	hsa-miR-204	+	+		+	
Q96A65	secretory protein SEC8	up	↑ -1.295874	hsa-miR-24			+		
O60610	diaphanous homolog 1 (Drosophila)	up	→ -8.746385	hsa-miR-198				+	
Q92886	neurogenin 3	up	↓ -12.7369	hsa-miR-204				+	

Grade II Stage III

SP ID	Target Name	Regulation	Fold Change	miRNA	Apoptosis	Cancer	Cell Cycle	Ptn Kinases
P25912	MAX protein	down	↑ 21.934555	hsa-miR-105		+		+
Q14566	MCM6 minichromosome maintenance deficient 6 (M	down	↓ 9.7958231	hsa-miR-96	+		+	
O43913	origin recognition complex, subunit 5-like (yeast)	down	↓ 6.9882195	hsa-miR-182			+	
P14923	junction plakoglobin	down	↑ -1.669324	hsa-miR-16		+		+
P51452	dual specificity phosphatase 3 (vaccinia virus phosph	up	↓ -6.181089	hsa-miR-211				+
P17964	RAP2B, member of RAS oncogene family	up	↑ -5.187142	hsa-miR-133b			+	
P54257	huntingtin-associated protein 1 (neuroan 1)	up	↑ -5.409039	hsa-miR-326				

Grade III Stage I

SP ID	Target Name	Regulation	Fold Change	miRNA	Apoptosis	Cancer	Cell Cycle	Ptn Kinases	
Q14016	scavenger receptor class B, member 1	down	↑ 21.93455	hsa-miR-105					
Q05086	ubiquitin protein ligase E3A (human papilloma virus E6-associated protein, Angelman syndrome)	down	↑ 11.2731	hsa-miR-141			+	+	
Q15701	dynein, cytoplasmic, light polypeptide 1	down	↑ 9.795823	hsa-miR-96			+		↓ -100
P20936	RAS p21 protein activator (GTPase activating protein) 1	down	↑ 6.98822	hsa-miR-182			+		
Q13617	cullin 2	down	↑ 6.98822	hsa-miR-182			+		
P28347	TEA domain family member 1 (SV40 transcriptional enhancer factor)	down	↑ 2.82262	hsa-miR-135a		+			
P35225	interleukin 13	down	↑ 2.470598	hsa-miR-98				+	
P25445	tumor necrosis factor receptor superfamily, member 6	down	↑ 2.470598	hsa-miR-98	+			+	
O75899	G protein-coupled receptor 51	down	↑ 2.470598	hsa-miR-98				+	↑ 100
P41002	cyclin F	down	↑ 2.470598	hsa-miR-98		+			
Q16543	CDC37 cell division cycle 37 homolog (S. cerevisiae)	down	↑ 2.368661	hsa-miR-155			+		
Q14289	PTK2B protein tyrosine kinase 2 beta	down	↑ 1.226652	hsa-miR-130b				+	
Q92673	sortilin-related receptor, L(DLR class) A repeats-containing	down	↓ -1.026431	hsa-miR-136					
P09601	heme oxygenase (decycling) 1	down	↓ -1.295874	hsa-miR-24					
P49959	MRE11 meiotic recombination 11 homolog A (S. cerevisiae)	down	↓ -2.124478	hsa-miR-197			+		
P26038	moesin	up	↓ -6.895061	hsa-let-7c					
P51531	SWI/SNF related, matrix associated, actin dependent regulator of chromatin, subfamily a, member 2	up	↓ -6.884174	hsa-miR-335				+	
P19634	solute carrier family 9 (sodium/hydrogen exchanger), isoform 1 (antiporter, Na+/H+, amiloride sensitive)	up	↓ -6.884174	hsa-miR-335					
P08247	synaptophysin	up	↓ -5.753737	hsa-miR-412					
Q92854	sema domain, immunoglobulin domain (Ig), transmembrane domain (TM) and short cytoplasmic domain, (semaphorin) 4D	up	↓ -5.130382	hsa-miR-215		+			
O95980	reversion-inducing-cysteine-rich protein with kazal motifs	up	↓ -4.16958	hsa-miR-302a		+	+		
P25963	nuclear factor of kappa light polypeptide gene enhancer in B-cells inhibitor, alpha	up	↓ -3.19402	hsa-miR-381				+	
P98082	disabled homolog 2, mitogen-responsive phosphoprotein (Drosophila)	up	↓ -2.908485	hsa-miR-150					
P07355	annexin A2	up	↓ -2.884268	hsa-miR-10b		+			
Q07820	myeloid cell leukemia sequence 1 (BCL2-related)	up	↓ -2.495869	hsa-miR-320	+	+	+		
P55060	CSE1 chromosome segregation 1-like (yeast)	up	↑ 1.492977	hsa-miR-19b	+		+		

Grade III Stage III

SP ID	Target Name	Regulation	Fold Change	miRNA	Apoptosis	Cancer	Cell Cycle	Ptn Kinases	
P21579	synaptotagmin I	down	↑ 10.62742	hsa-miR-429		+			↓ -100
Q9NR09	baculoviral IAP repeat-containing 6 (apollon)	down	↑ 9.795823	hsa-miR-96	+				
O43815	striatin, calmodulin binding protein	down	↑ 7.347348	hsa-miR-200a		+			
Q13617	cullin 2	down	↑ 6.98822	hsa-miR-182			+		↑ 100
Q9NNW1	protein phosphatase 1, regulatory (inhibitor) subunit 1B (dopamine and cAMP regulated phosphoprotein, DARPP-32)	down	↑ 5.352226	hsa-miR-302a*		+			
Q13017	Rho GTPase activating protein 5	down	↑ 5.352226	hsa-miR-302a*				+	
Q15149	plectin 1, intermediate filament binding protein 500kDa	down	↑ 2.973811	hsa-miR-7		+			
Q9Y2J0	likely ortholog of mouse rabphilin 3A	down	↓ -1.273598	hsa-miR-221					
P49418	amphiphysin (Stiff-Man syndrome with breast cancer 128kDa autoantigen)	down	↓ -2.107533	hsa-miR-134					
P13667	protein disulfide isomerase related protein (calcium-binding protein, intestinal-related)	down	↓ -2.469643	hsa-miR-214				+	
O15066	kinesin family member 3B	up	↓ -12.23899	hsa-miR-373		+			
Q07960	Rho GTPase activating protein 1	up	↓ -6.895061	hsa-let-7c		+	+		
P55210	caspase 7, apoptosis-related cysteine protease	up	↓ -6.895061	hsa-let-7c	+		+		
Q9UPH5	dynamin 2	up	↓ -2.469643	hsa-miR-214		+			
P35659	DEK oncogene (DNA binding)	up	7.347348	hsa-miR-200a	+	+	+		

Table S3 : MicroRNAs that are common between Human and mouse breast cancer

						GR2	GR2	GR2	GR2	GR3	GR3
Rodent miRs	Rod miRs	ER+ve	ER-ve	GR2	GR3	STGI	STGII	STGIII	STGI	STGII	STGIII
hsa-miR-23a*	-3.914	-18.11	-6.602	-11.48	-7.728	-15.82	-3.412	-20.74			
hsa-miR-93*	1.0125			1.4613	-1.045	1.1188	-1.008	1.9273	1.742	1.1667	-1.63
hsa-miR-183*	1.1524	3.6119	-4.067	4.5237	2.5321	4.1835	2.8549	7.1114	3.6443	3.9366	1.9082
hsa-miR-376a*	1.7277					1.1753	-1.879	-4.925	-3.8	-1.506	-1.322
hsa-miR-140-3p	2.1765	-7.219	-3.209	-8.8	-4.962	-11.38	-14.91	-5.282	-5.239	-3.819	-4.601
hsa-miR-27a*	2.4901								1.3726	2.4449	-1.079
hsa-miR-30c-1*	8.4769	-68.72	-265.7	-22.63	-32.72				-458.8	-13.16	-32.9
hsa-miR-200b	8.733	4.1227	-5.435	5.0968	3.9268	8.9987	1.1016	7.7534	3.5953	2.203	7.3991
hsa-miR-875-5p	10.361	-3.397	-1.098			-1.058	-5.117	-6.002			
hsa-miR-99b*	11.329			-2.031	-2.037	-4.393	-2.776	-1.07			
hsa-miR-299-5p	14.888	-7.315	-10.78	-7.91	-4.516				-37.6	-4.486	-1.076
hsa-miR-154*	16.202	-7.498	-4.143	-5.809	-5.834				-9.909	-3.761	-2.272
hsa-miR-378	23.567	-11.88	-4.491	-12.2	-8.046	-17.01	-10.09	-12.9	-6.693	-6.23	-7.586
hsa-miR-324-3p	42.257	-2.733	-1.664			-1.795	-8.375	-1.583	-2.976	-3.109	-1.245
hsa-miR-28-3p	59.434					-2.515	-10.1	-1.668	-2.46	-2.199	1.0904
hsa-miR-136	65.303					-6.321	-3.232	-1.397			
hsa-miR-200c	109.59	2.6029	-3.752	2.6072	2.7146	1.5012	-1.038	5.6292			
hsa-miR-143	124.19	-4.221	-5.115	-3.718	-5.115	-4.408	-8.091	-2.691	-7.327	-4.411	-1.657
hsa-miR-136*	306.56	2.4419	-3.39								

Table S4: The list of highly significant microRNAs in IDC with different subtypes and grades that could be novel biomarker for diagnosis and prognosis of IDC patients.

Table. A. MicroRNAs significantly up/down regulated in ER+ve

MicroRNAs	Sequence	Accession Id	Fold
hsa-miR-623	AUCCCUUGCAGGGGCUGUUGGGU	MIMAT0003292	↓ -40.86
hsa-miR-302d	UAAGUGCUUCCAUGUUUGAGUGU	MIMAT0000718	↓ -34.48
hsa-miR-562	AAAGUAGCUGUACCAUUUGC	MIMAT0003226	↓ -31.96
hsa-miR-224	CAAGUCACUAGUGGUUCCGUU	MIMAT0000281	↓ -17.46
hsa-miR-452	AACUGUUUGCAGAGGAAACUGA	MIMAT0001635	↓ -17.33
hsa-miR-522	AAAAUGGUUCCCUUUAGAGUGU	MIMAT0002868	↓ -15.14
hsa-miR-124	UAAGGCACGCGGUGAAUGCC	MIMAT0000422	↓ -12.28
hsa-miR-516a-5p	UUCUCGAGGAAAGAAGCACUUUC	MIMAT0004770	↓ -11.8
hsa-miR-521	AACGCACUUCCCUUUAGAGUGU	MIMAT0002854	↓ -10.9
hsa-miR-627	GUGAGUCUCUAAGAAAAGAGGA	MIMAT0003296	↓ -4.234
hsa-miR-650	AGGAGGCAGCGCUCUCAGGAC	MIMAT0003320	↓ -3.255
hsa-miR-205	UCCUUCAUUCCACCGGAGUCUG	MIMAT0000266	↓ -3.148
hsa-miR-605	UAAAUCCCAUGGUGCCUUCUCCU	MIMAT0003273	↑ 13.312
hsa-miR-375	UUUGUUCGUUCGGCUCGCGUGA	MIMAT0000728	↑ 13.609
hsa-miR-190b	UGAUAUGUUUGAUAUUGGGUU	MIMAT0004929	↑ 40.58

***p values 0.01-2.40E-14**

Table. B. MicroRNAs significantly up/down regulated in ER-ve

MicroRNAs	Sequence	Accession Id	Fold
hsa-miR-887	GUGAACGGGCGCCAUCCCGAGG	MIMAT0004951	↓ -10.91
hsa-miR-126*	CAUUAUUACUUUUGGUACGCG	MIMAT0000444	↓ -3.718
hsa-miR-188-5p	CAUCCCUUGCAUGGUGGAGGG	MIMAT0000457	↑ 2.6007
hsa-miR-210	CUGUGCGUGUGACAGCGGCUGA	MIMAT0000267	↑ 3.6748
hsa-miR-20a	UAAAGUGCUUAUAGUGCAGGUAG	MIMAT0000075	↑ 3.8147
hsa-miR-31	AGGCAAGAUGCUGGCAUAGCU	MIMAT0000089	↑ 4.1211
hsa-miR-187	UCGUGUCUUGUGUUGCAGCCGG	MIMAT0000262	↑ 4.6737
hsa-miR-301b	CAGUGCAAUGAUAUUGUCAAAGC	MIMAT0004958	↑ 5.6936
hsa-miR-142-3p	UGUAGUGUUUCCUACUUUAUGGA	MIMAT0000434	↑ 5.9133
hsa-miR-18a	UAAGGUGCAUCUAGUGCAGAUAG	MIMAT0000072	↑ 6.9885
hsa-miR-137	UUAUUGCUUAAGAAUACGCGUAG	MIMAT0000429	↑ 7.8731
hsa-miR-9	UCUUUGGUUAUCUAGCUGUAUGA	MIMAT0000441	↑ 8.1181
hsa-miR-135b*	AUGUAGGGCUAAAAGCCAUGGG	MIMAT0004698	↑ 8.6834
hsa-miR-934	UGUCUACUACUGGAGACACUGG	MIMAT0004977	↑ 15.642

***p values 0.01-0.00098**

Table. C. MicroRNAs significantly up/down regulated in Grade 2

MicroRNAs	Sequence	Accession Id	Fold
hsa-miR-143*	GGUGCAGUGCUGCAUCUCUGGU	MIMAT0004599	↓ -78.87
hsa-miR-361-3p	UCCCCCAGGUGUGAUUCUGAUUU	MIMAT0004682	↓ -20.76
hsa-miR-129-3p	AAGCCCUUACCCCAAAAAGCAU	MIMAT0004605	↓ -10.96
hsa-miR-561	CAAAGUUUAAGAUCCUUGAAGU	MIMAT0003225	↓ -4.985
hsa-miR-548b-5p	AAAAGUAAUUGUGGUUUUGGCC	MIMAT0004798	↓ -4.389
hsa-miR-627	GUGAGUCUCUAAGAAAAGAGGA	MIMAT0003296	↓ -4.37
hsa-miR-92a-1*	AGGUUGGGAUCGGUUGCAAUGCU	MIMAT0004507	↓ -1.841
hsa-miR-93*	ACUGCUGAGCUAGCACUUCCCG	MIMAT0004509	↑ 1.4613
hsa-miR-571	UGAGUUGGCCAUCUGAGUGAG	MIMAT0003236	↑ 2.2382
hsa-miR-7-1*	CAACAAAUCACAGUCUGCCAUA	MIMAT0004553	↑ 2.4298
hsa-miR-26a-2*	CCUAUUCUUGAUUACUUGUUUC	MIMAT0004681	↑ 2.9293
hsa-miR-449b	AGGCAGUGUAUUGUUAGCUGGC	MIMAT0003327	↑ 10.184
hsa-miR-449a	UGGCAGUGUAUUGUUAGCUGGU	MIMAT0001541	↑ 16.081

***p values 0.01-9.09E-06**

Table. D. MicroRNAs significantly up/down regulated in Grade 3

MicroRNAs	Sequence	Accession Id	Fold
hsa-miR-195*	CCAAUAUUGGCUGUGCUGCUCC	MIMAT0004615	↓ -230.2
hsa-miR-567	AGUAUGUUCUUCCAGGACAGAAC	MIMAT0003231	↓ -11.58
hsa-miR-29c*	UGACCGAUUUCUCCUGGUGUUC	MIMAT0004673	↓ -4.963
hsa-miR-30e*	CUUUCAGUCGGAUGUUUACAGC	MIMAT0000693	↓ -3.294
hsa-miR-30a*	CUUUCAGUCGGAUGUUUGCAGC	MIMAT0000088	↓ -3.101
hsa-miR-29b-2*	CUGGUUUCACAUGGUGGCUUAG	MIMAT0004515	↓ -2.688
hsa-miR-135b	UAUGGCUUUUCAUUCCUAUGUGA	MIMAT0000758	↑ 6.4166
hsa-miR-767-5p	UGCACCAUGGUUGUCUGAGCAUG	MIMAT0003882	↑ 101.54

***p values 0.01-8.520.9.09E-07**

Table. E. MicroRNAs significantly up/down regulated in Grade 2 Stage I

MicroRNAs	Sequence	Accession Id	Fold
hsa-miR-874	CUGCCCUGGCCCGAGGGACCGA	MIMAT0004911	↓ -86.32
hsa-miR-487a	AAUCAUACAGGGACAUCCAGUU	MIMAT0002178	↓ -41.49
hsa-miR-655	AUAAUACAUGGUUAACCUCUUU	MIMAT0003331	↓ -13.23
hsa-miR-30d*	CUUUCAGUCAGAUGUUUGCUGC	MIMAT0004551	↓ -6.504
hsa-miR-136	ACUCCAUUUGUUUUGAUGAUGGA	MIMAT0000448	↓ -6.321

*p values 0.0067-0.003

Table. F. MicroRNAs significantly up/down regulated in Grade 2 Stage II

MicroRNAs	Sequence	Accession Id	Fold
hsa-miR-509-5p	UACUGCAGACAGUGGCAAUCA	MIMAT0004779	↓ -34.51
hsa-miR-365	UAAUGCCCCUAAAAAUCCUUAU	MIMAT0000710	↓ -8.812
hsa-miR-92a	UAUUGCACUUGUCCCGGCCUGU	MIMAT0000092	↓ -8.118
hsa-miR-130a	CAGUGCAAUGUUAAAAGGGCAU	MIMAT0000425	↓ -8.054
hsa-miR-532-3p	CAUGCCUUGAGUGUAGGACCGU	MIMAT0002888	↓ -6.647
hsa-miR-30b	UGUAAACAUCCUACACUCAGCU	MIMAT0000420	↓ -6.621
hsa-miR-140-5p	CAGUGGUUUUACCCUAUGGUAG	MIMAT0000431	↓ -6.466
hsa-miR-362-5p	AAUCCUUGGAACCUAGGUGUGAGU	MIMAT0000705	↓ -6.387
hsa-miR-221	AGCUACAUUGUCUGCUGGGUUUC	MIMAT0000278	↓ -6.379
hsa-let-7e	UGAGGUAGGAGGUUGUAUAGUU	MIMAT0000066	↓ -6.095
hsa-miR-324-5p	CGCAUCCCCUAGGGCAUUGGUGU	MIMAT0000761	↓ -6.073
hsa-let-7a	UGAGGUAGUAGGUUGUAUAGUU	MIMAT0000062	↓ -5.936
hsa-let-7d	AGAGGUAGUAGGUUGCAUAGUU	MIMAT0000065	↓ -5.833
hsa-miR-25	CAUUGCACUUGUCUCGGUCUGA	MIMAT0000081	↓ -5.692
hsa-miR-20b	CAAAGUGCUCAUAGUGCAGGUAG	MIMAT0001413	↓ -5.262
hsa-miR-491-5p	AGUGGGGAACCCUUCCAUGAGG	MIMAT0002807	↓ -4.982
hsa-miR-99b	CACCCGUAGAACCGACCUUGCG	MIMAT0000689	↓ -4.539
hsa-miR-345	GCUGACUCCUAGUCCAGGGCUC	MIMAT0000772	↓ -4.094

*p values 0.01-0.00055

Table. G. MicroRNAs significantly up/down regulated in Grade 2 Stage III

MicroRNAs	Sequence	Accession Id	Fold
hsa-miR-661	UGCCUGGGUCUCUGGCCUGCGCGU	MIMAT0003324	↓ -72.57
hsa-miR-376a*	GUAGAUUCUCCUUCUAUGAGUA	MIMAT0003386	↓ -4.925
hsa-miR-625*	GACUAUAGAACUUUCCCCCUCA	MIMAT0004808	↑ 1.674
hsa-miR-766	ACUCCAGCCCCACAGCCUCAGC	MIMAT0003888	↑ 1.7898
hsa-miR-200c	UAAUACUGCCGGGUAAUGAUGGA	MIMAT0000617	↑ 5.6292
hsa-miR-598	UACGUCAUCGUUGUCAUCGUCA	MIMAT0003266	↑ 6.1447
hsa-miR-135a	UAUGGCUUUUUAUUCCUAUGUGA	MIMAT0000428	↑ 9.0314
hsa-miR-184	UGGACGGAGAACUGAUAAGGGU	MIMAT0000454	↑ 22.902

*p values 0.01-0.00037

Table. H. MicroRNAs significantly up/down regulated in Grade 3 Stage I

MicroRNAs	Sequence	Accession Id	Fold
hsa-miR-654-5p	UGGUGGGCCGCAGAACAUGUGC	MIMAT0003330	↓ -61.19
hsa-miR-154	UAGGUUAUCCGUGUUGCCUUCG	MIMAT0000452	↓ -55.32
hsa-miR-499-5p	UUAAGACUUGCAGUGAUGUUU	MIMAT0002870	↓ -42.64
hsa-miR-299-5p	UGGUUUACCGUCCCACAUACAU	MIMAT0002890	↓ -37.6
hsa-miR-431	UGUCUUGCAGGCCGUCAUGCA	MIMAT0001625	↓ -16.2
hsa-miR-381	UAUACAAGGGCAAGCUCUCUGU	MIMAT0000736	↓ -13.55
hsa-miR-337-5p	GAACGGCUUCAUACAGGAGUU	MIMAT0004695	↓ -13.22
hsa-miR-369-5p	AGAUCGACCGUGUUAUAUUCGC	MIMAT0001621	↓ -10.5
hsa-miR-154*	AAUCAUACACGGUUGACCUAUU	MIMAT0000453	↓ -9.909
hsa-miR-615-5p	GGGGGUCCCCGGUGCUCGGAUC	MIMAT0004804	↓ -8.392
hsa-miR-542-5p	UCGGGGAUCAUCAUGUCACGAGA	MIMAT0003340	↓ -7.186
hsa-miR-539	GGAGAAAUUAUCCUUGGUGUGU	MIMAT0003163	↓ -4.765
hsa-miR-379	UGGUAGACUAUGGAACGUAGG	MIMAT0000733	↓ -3.924
hsa-miR-376a	AUCAUAGAGGAAAAUCCACGU	MIMAT0000729	↓ -3.8
hsa-miR-19a*	AGUUUUGCAUAGUUGCACUACA	MIMAT0004490	↑ 8.2999
hsa-miR-586	UAUGCAUUGUAUUUUUAGGUCC	MIMAT0003252	↑ 9.2991

*p values 0.01-6.98E-05

Table. I. MicroRNAs significantly up/down regulated in Grade 3 Stage II

MicroRNAs	Sequence	Accession Id	Fold
hsa-miR-760	CGGCUCUGGGUCUGUGGGGA	MIMAT0004957	↓ -5.391
hsa-let-7e*	CUAUACGGCCUCCUAGCUUUCC	MIMAT0004485	↓ -1.334
hsa-miR-30d	UGUAAACAUCCCCGACUGGAAG	MIMAT0000245	↓ -1.684
hsa-miR-27a*	AGGGCUUAGCUGCUUGUGAGCA	MIMAT0004501	↑ 1.3726
hsa-miR-941	CACCCGGCUGUGUGCACAUGUGC	MIMAT0004984	↑ 1.4397
hsa-miR-493*	UUGUACAUGGUAGGCUUUCAUU	MIMAT0002813	↑ 1.9123

*p values 0.0023-0.00085

Table. J. MicroRNAs significantly up/down regulated in Grade 3 Stage III

MicroRNAs	Sequence	Accession Id	Fold
hsa-miR-584	UUAUGGUUUGCCUGGGACUGAG	MIMAT0003249	-13.7
hsa-miR-193b*	CGGGGUUUUGAGGGCGAGAUGA	MIMAT0004767	-8.708
hsa-miR-200c*	CGUCUUACCCAGCAGUGUUUGG	MIMAT0004657	6.7748
hsa-miR-147b	GUGUGCGGAAAUGCUUCUGCUA	MIMAT0004928	12.91
